# Validation and Application of a PCR Primer Set to Quantify Fungal Communities in the Soil Environment by Real-Time Quantitative PCR

**DOI:** 10.1371/journal.pone.0024166

**Published:** 2011-09-08

**Authors:** Nicolas Chemidlin Prévost-Bouré, Richard Christen, Samuel Dequiedt, Christophe Mougel, Mélanie Lelièvre, Claudy Jolivet, Hamid Reza Shahbazkia, Laure Guillou, Dominique Arrouays, Lionel Ranjard

**Affiliations:** 1 INRA-Université de Bourgogne, UMR Microbiologie du Sol et de l'Environnement, CMSE, Dijon, France; 2 Université de Nice and CNRS UMR 6543, Laboratoire de Biologie Virtuelle, Centre de Biochimie, Parc Valose, Nice, France; 3 Platform GenoSol, INRA-Université de Bourgogne, CMSE, Dijon, France; 4 INRA Orléans - US 1106 InfoSol, Orleans, France; 5 DEEI-FCT, Universidade do Algarve, Campus de Gambelas, Faro, Portugal; 6 Université Pierre and Marie Curie and CNRS, UMR 7144, Adaptation et Diversité en Milieu Marin, Station Biologique de Roscoff, Roscoff, France; University of Wisconsin – Madison, United States of America

## Abstract

Fungi constitute an important group in soil biological diversity and functioning. However, characterization and knowledge of fungal communities is hampered because few primer sets are available to quantify fungal abundance by real-time quantitative PCR (real-time Q-PCR). The aim in this study was to quantify fungal abundance in soils by incorporating, into a real-time Q-PCR using the SYBRGreen® method, a primer set already used to study the genetic structure of soil fungal communities. To satisfy the real-time Q-PCR requirements to enhance the accuracy and reproducibility of the detection technique, this study focused on the 18S rRNA gene conserved regions. These regions are little affected by length polymorphism and may provide sufficiently small targets, a crucial criterion for enhancing accuracy and reproducibility of the detection technique. An *in silico* analysis of 33 primer sets targeting the 18S rRNA gene was performed to select the primer set with the best potential for real-time Q-PCR: short amplicon length; good fungal specificity and coverage. The best consensus between specificity, coverage and amplicon length among the 33 sets tested was the primer set FR1 / FF390. This *in silico* analysis of the specificity of FR1 / FF390 also provided additional information to the previously published analysis on this primer set. The specificity of the primer set FR1 / FF390 for *Fungi* was validated *in vitro* by cloning - sequencing the amplicons obtained from a real time Q-PCR assay performed on five independent soil samples. This assay was also used to evaluate the sensitivity and reproducibility of the method. Finally, fungal abundance in samples from 24 soils with contrasting physico-chemical and environmental characteristics was examined and ranked to determine the importance of soil texture, organic carbon content, C∶N ratio and land use in determining fungal abundance in soils.

## Introduction

Soil plays a crucial role in determining the rates and the diversity of ecosystem processes. Indeed, soil houses very large quantities of microorganisms with enormous biodiversity [1]–[4], resulting in numerous biological interactions and ecological processes. To date, most studies have focused on soil bacteria and analyzed their diversity [5], ecology [6]–[8], or role in biogeochemical cycles [9], [10]. Despite the important role of fungi in ecosystem functioning (*e.g.* nutrient and C cycling) and their huge biodiversity (1.5 million species; [11]), studies of soil fungal communities represent only about 30% of the total investigations of soil microbial communities reported in the literature. In the context of molecular ecology, this trend may be observed because fewer molecular tools are available for the *in situ* characterization of soil fungi [12], the genetic sequence databases for soil fungi are smaller than those for soil bacteria, and also because fewer groups are working on soil fungi. However, the need to develop new tools to improve our ability to characterize the diversity and abundance of soil fungal communities has been highlighted by the rapid evolution from descriptive to quantitative approaches in microbial ecology. An absolute quantification of soil fungal communities could i) provide a simple bio-indicator for evaluating the impact of human activities on soil; ii) reveal the relative importance of soil fungi, as compared to bacteria, in the total microbial biomass. This result could also be combined to the quantification of specific fungal phyla to estimate their relative abundance. Finally, this would lead to a better understanding of the role of fungi in soil biological functioning.

Real-time quantitative PCR (real-time Q-PCR) has recently become a valuable molecular tool for quantifying indigenous organisms in environmental samples directly from environmental DNA extracts. This method is powerful, accurate and culture-independent. Different taxonomic levels can be attained by targeting different regions in the genome [13], [14] (*e.g.* “broad” taxonomic resolution with targets located in rrs genes, and finer taxonomic resolution with targets located in more variable regions like the Internal Transcribed Spacer (ITS)). The real-time Q-PCR method has been used successfully to measure total bacterial abundance [14] and the abundance of bacteria involved in the nitrogen cycle in soils [13]. In addition, the suitability of the method for quantifying soil fungal communities has been demonstrated *in vitro*. Raidl et al. [15] demonstrated a linear relationship between the number of copies of the ITS region detected by real-time Q-PCR and the hyphal length of *Piloderma croceum*, an ectomycorrhizal fungus. Nevertheless, real-time Q-PCR still needs to be improved for the study of soil fungal communities. Indeed, when this approach was used in different studies to target the ITS region in fungi in environmental samples [16]–[23], the reproducibility and accuracy of the real-time Q-PCR measurements of absolute fungal abundance in soil samples were hampered by the length of the ITS region, together with its high length polymorphism and potential resulting taxonomic bias [24]. The reproducibility and accuracy of the method constitute strong limitations in ecological studies of soil fungal communities [25]. These are largely determined by the length of the amplicon produced [14], [25]: short amplicon enhance the accuracy and the reproducibility of the method. To overcome these limitations and enhance the reproducibility of the method for soil fungal communities, some studies focused on the 18S rRNA gene region [26]–[28]. This region was chosen because it contains conserved regions with only slight length polymorphism and because sequence polymorphism is not a limiting factor in the real-time Q-PCR approach. However, limitations related to the length of the targeted region [28] or to the specificity of the primer set [26], [27] were still encountered. This highlighted the need to identify other primer sets suitable for use with real-time Q-PCR.

Our aim was therefore to extend the use of a primer set commonly involved in the characterization of soil fungal community composition in the literature, with quantification of the soil fungal community by real-time Q-PCR. The 18S rRNA gene was chosen as the target gene because, conversely to the ITS region, it contains conserved regions unaffected by length polymorphism. To identify and evaluate the suitable primer set, a three step procedure was chosen. First, 33 primer sets targeting the 18S rRNA gene were compared *in silico* for the length of the amplicon, so as to ensure good accuracy and reproducibility of the detection technique. This allowed the selection of a subset of primer sets that produced shorter amplicons than the primer set nu-SSU-0817/nu-SSU-1196 [26], already used in combination with real-time Q-PCR approach. Among this subset, the primer sets were tested for specificity for *Fungi* against a theoretical optimal primer (fully specific of *Fungi*) to identify the best sets. These best sets were then compared in details to one another for their specificity and coverage for *Fungi*. This allowed the selection of the more relevant primer set corresponding to the best consensus between fungal specificity, fungal coverage and amplicon length: FR1/FF390 (targeted gene: 18S rRNA gene, target region length: *c.a.* 350 bp). This primer set was developed a decade ago by Vainio and Hantula [29] for the analysis of wood-inhabiting fungi by Denaturating Gradient Gel Electrophoresis (DGGE), and is frequently used in combination with DGGE to analyze fungal community composition in soil (*e.g.* [30]–[32]). Second, a real time Q-PCR run was performed using the primer set FR1 / FF390 on five independent soil samples with serial dilutions of template DNA. The resulting amplicons were used to validate the fungal specificity of the primer set FR1 / FF390 *in vitro* through a cloning - sequencing approach and the real-time Q-PCR data from this run were used to evaluate method sensitivity and reproducibility. Third, the method was applied to 24 soil samples originating from different physico-chemical conditions and subjected to various land-use practices (forests, grasslands and croplands) to evaluate the ecological potential of this tool and rank the influence of soil properties and land-use practices on soil fungal abundance.

## Results and Discussion

### *In Silico* Selection and Validation of the relevant Primer Set

65 unique primers located in the 18S rRNA gene were extracted from the literature and from the AFTOL primer database. They were analyzed *in silico* as 33 primer sets (Data S1). The relevant primer set was selected as the best consensus between fungal specificity, fungal coverage and the length of the amplicon. The 33 primer sets were first discriminated for the length of the amplicon produced (Figure S1) which ranged from 135 bp (±9 bp) to 523 bp (±3 bp). This lead to the selection of a subset of 23 primer sets that were tested for their fungal specificity against a theoretical optimal primer set (fully fungal specific) through an ascendant hierarchical classification (Data S1) which produced five significant clusters (*P*<0.05) of primer sets (Figure S2). The best primer sets were clustered with the theoretical optimal primer and were: nu-SSU-0817/nu-SSU-1196, FF390/FR1, nssu897R/nu-SSU-1196 and nssu1088R/SR2.

**Figure 1:**
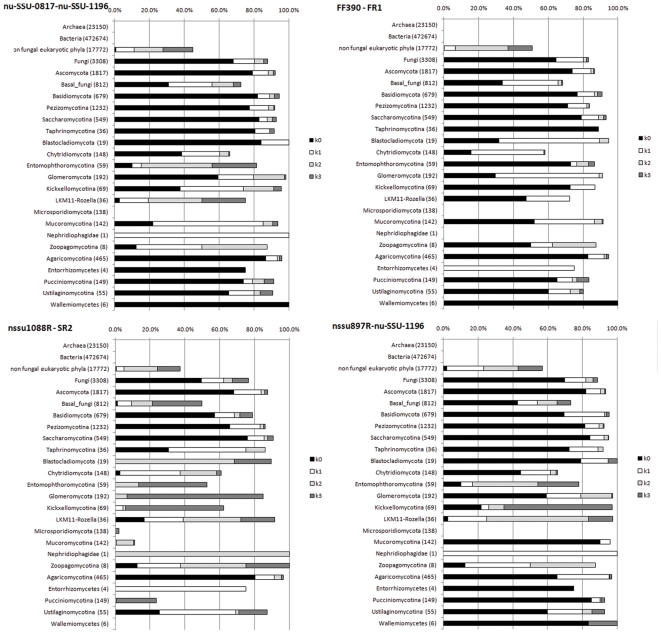
*In silico* comparison of the primer sets nu-SSU-0817/nu-SSU-1196, FR1/FF390, nssu1088R-SR2 and nssu897R-nu-SSU-1196 for their fungal-specificity. For each primer set, k mismatches (0 to 3) were allowed in the *in silico* analysis to test the specificity of the original primer set (k = 0) and its potential sequence improvement (k = 1 to 3). For each graph, each bar represents the hit frequency (%) of the primer set for the selected phylum with: k = 0: black, k = 1: dark grey, k = 2: white, k = 3: light grey. The number of sequences available for a phylum is indicated in brackets. Detailed hit frequencies are provided in Table S1.

We detail below the comparison between these 4 “best” primer sets considering non fungal phyla and the *Fungi* kingdom (Figure 1). Similarities with 0 to 3 mismatches were evaluated in the comparison of the four “best” sets, but only the 0 mismatch analysis, *i.e.* the original primer sets, was examined for specificity and coverage of the *Fungi* kingdom. Analyses involving 1 to 3 mismatches were then examined to test the possibility of improving the primer set sequences and enhancing fungal detection without diminishing the specificity of each primer set for *Fungi*.

The *in silico* analysis indicated that only *Eukaryota* sequences were detected by the four “best” sets (Table S1). Except nssu1088R / SR2, the primer sets had a relatively good coverage of *Fungi* kingdom (64.5% to 69.8%) but also punctual matches with some non-fungal groups. FR1 / FF390 is the set that matched the fewer non fungal groups: *Choanoflagellida* (<0.8%, *Choanoflagellida clade-2*), *Mesomycetozoa* (10.8%, *Ichthyosporea* and *Nuclearia* sequences), and some *Metazoa* (<0.3%, *Cnidaria* and *Porifera* sequences); which was not documented in the literature [30], [32]–[34]. Except for *Nuclearia*, none of the non-fungal groups matched by the primer set are found in soils. The FR1 / FF390 primer set would thus be relevant for a robust and specific detection of the soil fungal community. In comparison, the other primer sets matched these groups at similar or higher levels (*e.g. Choaniflagellida, Metazoa*), and additional non-fungal groups (*e.g. Cryptophyta*, *Alveolata*, *Oxymonadida*, *Stramenopiles*) potentially found in soils. This lead to the conclusion that FR1 / FF390 primer set was more fungal specific than the other 3 primer sets.

At the fungal level, major phyla (*Ascomycota* and *Basidiomycota*) were very efficiently detected by the primer sets FR1 / FF390 and nssu897R / nu-SSU-1196 (ca. 75% to 80% for both phyla and both primer sets). The other sets presented smaller coverage of each group (nssu-1088R/SR2) or a disequilibrium between the two groups (nu-SSU-0817/nu-SSU-1196). The different sub-groups of *Ascomycota* and *Basidiomycota* were also well-covered (coverage ranging from 60% to 87%). None of the four primer sets had a very large coverage of the basal fungal lineages (coverage ranging between 31.0% and 42.6%, except nssu1088R / SR2 with 1.4%). This was mainly determined by the low detection of some basal fungal lineages (*Chytridiomycota*, *Glomeromycota* and *Blastocladiales*). The poorer detection of basal fungal lineages, in relation to other fungal groups, is in agreement with the literature [33]–[35] but shows that the FR1/FF390 and nssu897R/nu-SSU-1196 primer sets are able to take these groups into account, if only partially. Nevertheless, nssu897R/nu-SSU-1196 remained less fungal specific than FR1 / FF390.

Finally, this analysis showed that the primer set FR1 / FF390 was the best consensus between a short amplicon and a good specificity and coverage of *Fungi*. The original FR1 / FF390 primer set seems better suited for combination with real-time Q-PCR among the sets tested *in silico*. Indeed, it is specific for *Fungi*, matches every major fungal phylum and avoids technical limitations related to target length polymorphism. Even if FR1 / FF390 does not provide fully exhaustive coverage of the fungal kingdom, the non-covered phyla belong to basal fungal lineages that represent only 1.5–2.0% of the total number of fungal taxa identified to date in the Genbank database [36]. Therefore, soil fungal community abundance should only be slightly underestimated. In addition, the major fungal phyla that might strongly influence estimates of soil fungal abundance are largely and almost equally covered.

For the 4 “best” primer sets, the introduction of mismatches, *i.e.* degenerating primer set sequences into the *in silico* analysis, allowed the test of the potential improvement of their sequences. This effectively increased the hit frequency of the different fungal phyla from 70% to 95% (Figure 1) but decreased the fungal specificity of every primer set with the detection of additional non-fungal organisms to a large extent, particularly *Metazoa*, *Chlorophyta*, *Stramenopiles* or *Cercozoa*,. This does not constitute a good compromise for the real-time Q-PCR approach because non fungal sequences cannot be discarded by any post-processing method. Therefore, modifying the sequences in any primer set would have produced biased estimations of soil fungal abundance.

### *In Vitro* Evaluation of the FR1 / FF390 Primer Set

Five soils with contrasting texture, C and N contents, C∶N ratio and pH (Table 1), were first used to evaluate the sensitivity, efficiency and reproducibility of the method and thereby define the amount of DNA template to use in the real-time Q-PCR assay on the basis of this information. In a second step, the real-time Q-PCR products obtained directly from these five soils were then cloned and sequenced to validate the specificity of the FR1 / FF390 primer set.

**Table 1:** Physico-chemical characteristics of the soil samples used for *in vitro* validation of the FR1/FF390 primer set.

Site number	C_org_ NCaCO_3_P	K	pH_water_	Texture	C∶N
	g kg^−1^				%			
858	15.0	1.6	BD	0.08	1.5	7.1	Silt Clay	9.4
1012	9.7	1.1	BD	0.10	1.1	7.1	Silt Loam	8.9
1051	26.2	2.5	BD	0.04	3.6	5.4	Sandy Loam	10.4
1101	8.1	0.8	BD	0.09	1.6	6.4	Silt Loam	9.8
1143	60.0	3.0	BD	0.03	3.3	4.3	Sandy Loam	20.3

Texture was determined according to the USDA referential. C_org_: organic carbon content; N: total nitrogen content; P: available phosphorous; K: total potassium content. BD: below the detection threshold.

### Experimental Determination of the Sensitivity, Efficiency and Reproducibility of the Real-Time Q-PCR Approach

The threshold cycle (C_T_) was significantly and linearly related to the logarithm of the starting quantity of 18s rRNA gene copies on the standard curve (r^2^>0.99). This indicates that the method provides accurate estimates of the 18s rRNA gene copy number in pure DNA templates (standard template corresponding to the FR1 / FF390 target region derived from a pure culture of *Fusarium oxysporum* and cloned into PGEMT plasmid). The C_T_ of the no-templates assay was at least 3.3 cycles higher than that of the most diluted standard (3 10^2^ copies of 18S rRNA gene). The sensitivity of the method could therefore be set at 3 10^2^ copies of 18S rRNA gene per assay [37]. This detection limit is much lower than that defined for the nu-SSU-0817/nu-SSU-1196 primer set [27] and is within the range of detection limits defined for primer sets targeting the ITS region [17], [22].

**Figure 2:**
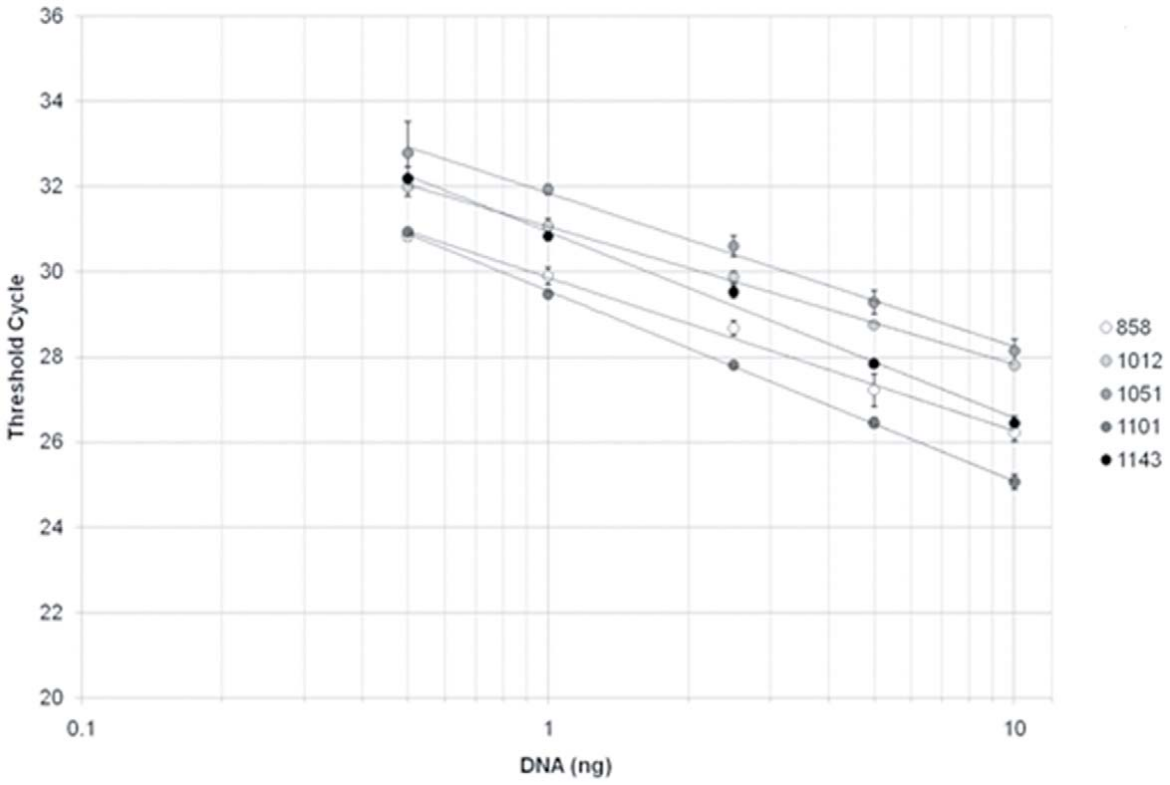
Threshold cycle against DNA quantity in the PCR mix for five soil DNA extracts with serial dilution. DNA quantities are represented in logarithmic scale and correspond to a serial dilution series (10 ng, 5 ng, 2.5 ng, 1 ng, and 0.5 ng). The linear regressions were highly significant (r^2^>0.99) for each soil type. The equations of the regression line were for each soil sample: 858: y = −3.61x+29.86; 1012: y = −3.24x+31.06; 1051: −3.60x+31.84; 1101: y = −4.47x+29.55; 1143: y = −4.37x+30.94.

The efficiency of the real-time Q-PCR method for soil DNA extracts was tested by serial dilution using DNA templates derived from five soil samples. For each soil sample, the relationship between the C_T_ value and the logarithm of the amount of DNA template in the PCR was linear and highly significant (r^2^>0.99, Figure 2, raw data are provided in Table S6). The PCR efficiencies (derived from the slope of the linear regression) differed from one soil sample to another and ranged between 67% and 103%. They were, however, within the ranges reported in the literature [17], [27] and in the same range as the efficiency derived from the standard curve (91%). The observed variations may be related to the different proportions of PCR inhibitors in the samples which vary according to the physico-chemical characteristics of soils. This was supported by the variations in PCR efficiency of each soil sample with DNA template concentration. PCR efficiency was close to the standard PCR efficiency for DNA template quantities of 1 ng to 2.5 ng, except for sample 1101 (73%).

**Figure 3:**
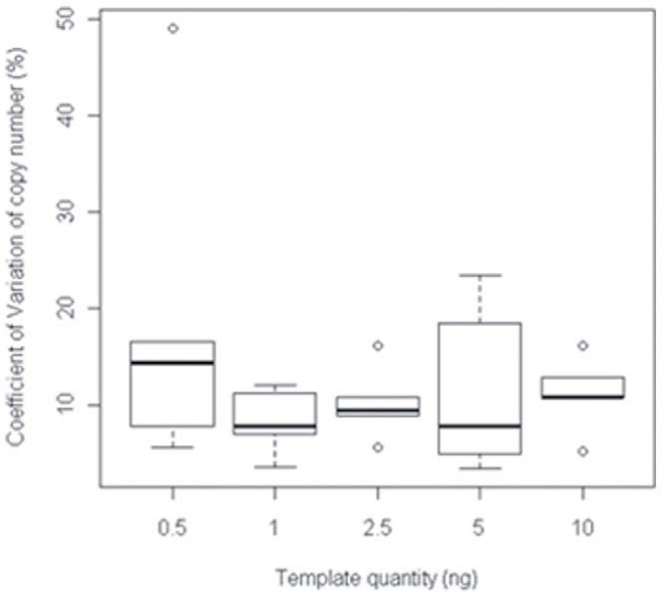
Variation coefficient of 18S rRNA gene copy number with DNA quantity in the PCR mix for five soil DNA extracts with serial dilution. The box limits represent the first and third quartiles of the variation coefficient (CV), the bold line represents the median and the error bars represent the standard deviation. Empty circles correspond to the minimum and maximum of the CV. The CV for each soil was determined from 3 independent measurements.

The reproducibility of the method for environmental samples was tested by calculating the coefficient of variation (CV) of C_T_ and of the number of copies of the 18S rRNA gene throughout the PCR assay for each DNA template quantity. For each soil, the C_T_ measurements were highly reproducible for a given DNA template quantity within an assay (CV<2.2%). The lowest ranges of variations in this CV were observed for DNA template quantities ranging from 1 ng to 2.5 ng. Nevertheless, the CV of the number of 18S rRNA gene copies estimated from the standard curve was much higher, ranging from 3% to 23% (Figure 3, except for sample 1051 for which the CV was 49% at the lowest DNA template quantity). This is within the range of CVs reported in the literature [37]–[39]. The higher CV obtained for the 18S rRNA gene copy number is probably related to error propagation during the conversion of C_T_ into copy number [37]. The CV values did not seem to be related to template quantity in the real-time Q-PCR mix for a given soil, but the ranges of variation of the 18S rRNA gene copy number were lowest (5% to 16%) for 2.5 ng of template DNA per PCR assay.

According to these results, and because the extractable DNA content of certain soil types may be very low, the DNA quantity in the PCR assay was set at 2.5 ng. This limited the error on the 18S rRNA gene copy number, which ranged from 5% to 16%. It also meant that the PCR efficiency of most templates was close to that of the standards, which ensured the accuracy of the method. The negative controls were below the detection limit set by the standard curve at *ca.* 10^2^ copies of 18S rRNA gene per PCR assay.

### Validation of Primer set Specificity

**Figure 4:**
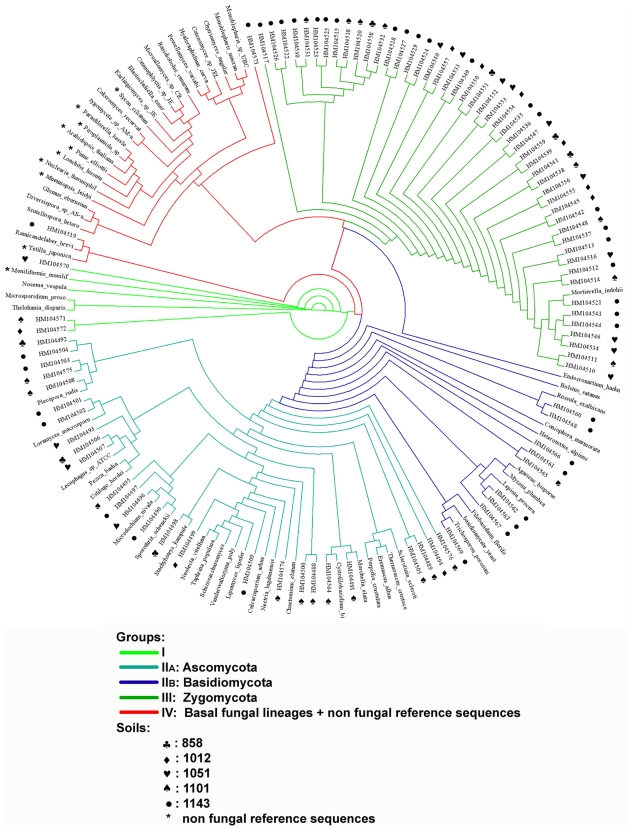
Distribution of clones obtained by the cloning-sequencing approach in the different fungal phyla. Symbols indicate the soil of origin of the clone: ♣: 858, ♦: 1012, ♡: 1051, ♠: 1101, •: 1143. *: Non fungal reference sequences. Genbank accession numbers of clones and their respective affiliation are provided in Table S2. Accession numbers of the non fungal reference sequences are provided in Table S3.

The real-time Q-PCR products obtained in the above-defined conditions, directly from the DNA of the five soils evaluated for sensitivity, efficiency and reproducibility, were cloned and sequenced to check the specificity of the primer set FR1 / FF390. NCBI-Blast was used for robust affiliation of the sequences. Only fungal sequences were identified (Table S2 for affiliation and accession numbers of the clone sequences). Most of the sequences were successfully affiliated and corresponded to fungal sequences. Six sequences were not completely affiliated but 3 of them were close to *Sordariomycetes* and the 3 others could only be related to eukaryotic fungal sequences. These sequences were aligned with reference sequences extracted from Genbank (Accession numbers in Table S3) to check that the clone sequences clustered according to their affiliation. Figure 4 presents the maximum parsimony dendrogram of the sequences of clones derived from the real time Q-PCR products. The clones did not cluster according to their soil of origin, so any potential bias due to manipulation was limited. The bootstrap values were not significant, due to the length of the sequences (317 to 360 bp), but the different phylogenetic methods tested (Neighbor Joining, Maximum Parsimony and Maximum Likelihood) produced similar clusters, which strengthened the analysis. In addition, the obtained clusters were in agreement with the fungal phylogeny presented in James et al. [36]: basal fungal lineages (groups I, III and IV) were discriminated from *Basidiomycota* (group II_B_) and *Ascomycota* (group II_A_). Nevertheless, the non-fungal reference sequences did not root the dendrogram and mainly formed a small group with the basal fungal lineages, to which they seem to be closest according to the phylogeny presented in James et al. [36]. The mix of non fungal sequences with basal fungal lineages was mainly determined by the *a priori* choice of reference sequences. Indeed, construction of the dendrogram of clone sequences alone by maximum parsimony method produced the same clusters and significant bootstrap values (Figure S3).

Clones affiliated to *Ascomycota* (22 clones (HM104488–HM104509) and 3 clones corresponding to fungal environmental samples related to *Sordariomycetes*, group II_A_ (HM104574–HM104576)), *Basidiomycota* (10 clones (HM104560–HM104569), group II_B_) or basal fungal lineages (50 clones of *Zygomycota* (HM104510–HM104559) and 1 clone of *Chytridiomycota* (HM104573) belonging to groups III and IV, respectively), clustered with their corresponding reference sequences. *Microsporidia* rooted the analysis together with 3 clones corresponding to fungal environmental samples (group I, (HM104570–HM104572)) which were not strictly related to any of the above groups. These observations confirmed the affiliation of the cloned sequences and the large spectrum of fungal phyla covered by the primer set, as indicated by our *in silico* analysis. This large spectrum of detection is in agreement with other studies that sequenced the DGGE bands derived by using the FR1 / FF390 primer set on soil DNA extracts [30], [33]–[35]. However, in our case, the clones belonging to basal fungal lineages were much more abundant than would be expected from our *in silico* analysis, the DGGE studies [34], [35] or mass sequencing studies [40], [41]. This was mainly due to high frequency of similar sequences in our clone library (*e.g.* 29 identical sequences of *Mucoromycotina*) constituting in only 6 OTUs at the 1% similarity level. Despite similar hit frequencies in the *in silico* analysis, *Ascomycota* sequences were also more abundant than *Basidiomycota* sequences in this study, which were distributed into 20 and 10 OTUs at the 99% similarity level, respectively. Differences in clone number may be related to the soil types selected to test the specificity of the FR1 / FF390 primer set. Indeed, most of the five soils had C∶N ratios lower than 10 and relatively high phosphorus (P) contents which could have increased the abundance of *Ascomycota* phyla rather than *Basidiomycota* phyla, according to the results of Lauber et al [19].

No *Nuclearia* sequences were detected in the clones, conversely to what was expected from the *in silico* analysis. This is in agreement with other studies involving this primer set in which only fungal sequences were detected [30], [33], [35], [42] and may be related to a relatively low abundance of *Nuclearia* (only 9 taxa recorded in the Genbank database).

No *Glomeromycota* was detected *in vitro* despite its potential amplification according to the *in silico* analysis. The absence of clones belonging to this particular fungal group may be explained by i) the low relative abundance of this group compared to major groups like *Ascomycota* or *Basidiomycota*; ii) the ecology of this group (mainly symbiotic and rhizospheric fungi according to Pivato et al. [43]) with regard to our soil samples (bulk soil containing mainly sporitic forms) which might limit their accessibility for cell lysis and DNA extraction, and/or iii) competition during amplification. In view of their important role in ecosystem functioning [9], a real time Q-PCR assay was run on DNA extracts from pure cultures of *Glomus versiforme*, *Glomus clarum*, *Glomus claroïdeum* , *Glomus geosporum* and on *Glomeromycota* rich rhizospheric soil DNA extracts [43]. The significant amplification of *Glomus sp.* (Table S4) demonstrated that the previously observed absence of *Glomeromycota* sequences was not related to the specificity of the FR1 / FF390 primer set, but more probably to their low abundance in bulk soil. To test this hypothesis, we applied our primer set and PCR conditions to rhizosphere soil of *Medicago truncatula* which is rich in *Glomeromycota* and detected a positive signal (Table S5; [44]), confirming our hypothesis.

We were able to conclude from the results of this *in vitro* analysis that the FR1 / FF390 primer set was fungi-specific and gave reliable results by amplifying the various fungal groups irrespective of their proportions in the soil samples. Indeed, the matching of particular fungal groups seems to depend mainly on their ecology, which determines their accessibility for DNA extraction, rather than on primer specificity.

### Ecological Validation of Real-Time Q PCR for Fungi

The developed method was applied to 24 independent soils of contrasting physico-chemical characteristics and land-use type (Table 2). The aim was to evaluate the combined use of FR1 / FF390 and real-time Q-PCR for ecological investigation by focusing on the determinism of the quantitative variation of fungal abundance in soil. The abundance of soil fungal communities estimated from the 18S rRNA gene copy number was not converted into “number of fungal cells per gram of soil” because many types of fungi are multinucleate cells with very variable numbers of nuclei per cell between species.

**Table 2:** Physico-chemical characteristics and land-use of the soil samples used for ecological validation of the FR1/FF390 primer set combined with a real-time Q-PCR approach.

Site number	C_org_**	N	CaCO_3_	P***	K	pH_water_	Texture	C∶N***	Land Use
	g kg^−1^				%				
634	19.0	1.8	190.0	0.1	0.5	8.3	Clay loam	10.6	Cropland
750	26.4	2.8	77.6	0.04	1.6	8.0	Clay	9.4	Cropland
854	10.5	0.9	BD	0.08	0.5	6.7	Loam	11.5	Cropland
914	34.4	3.6	56.8	0.1	1.5	7.9	Silt Clay	9.5	Cropland
917	18.8	1.8	BD	0.07	1.6	7.0	Silt Clay Loam	10.3	Cropland
968	11.5	1.2	BD	0.13	1.1	7.0	Silt Clay Loam	9.3	Cropland
1220	9.8	1.0	BD	0.04	3.5	5.9	Loamy Sand	9.9	Cropland
1224	25.6	2.5	6.0	0.03	1.7	7.8	Clay	10.1	Cropland
633	32.8	2.5	39.9	BD	0.4	7.7	Clay	13.0	Forest
693	24.3	1.6	BD	0.02	0.7	4.6	Loam	15.2	Forest
807	26.1	1.5	BD	BD	1.2	5.4	Silt Clay Loam	17.8	Forest
810	42.6	3.0	41.2	0.01	1.2	7.7	Silt Clay	14.3	Forest
857	85.4	6.0	239.0	0.02	0.7	8.0	Silt Clay Loam	14.3	Forest
910	99.5	6.4	47.2	0.02	1.2	7.4	Clay	15.5	Forest
1004	56.8	4.2	130.0	BD	1.4	7.8	Clay	13.6	Forest
1053	42.8	2.8	BD	0.02	2.4	5.0	Loam	15.1	Forest
907	25.0	2.4	BD	0.03	0.9	5.9	Loam	10.3	Grassland
963	23.7	2.3	BD	0.02	2.5	5.6	Loam	10.4	Grassland
965	42.8	4.5	BD	0.02	2.0	6.8	Silt Clay	9.4	Grassland
1095	29.2	2.9	1.3	0.02	1.8	6.9	Clay	10.0	Grassland
1099	21.8	2.1	106	0.04	2.0	8.1	Clay	10.2	Grassland
1146	9.9	0.9	BD	0.04	1.0	6.1	Silt Loam	10.6	Grassland
1182	17.1	1.9	BD	0.04	3.7	5.5	Sandy Loam	8.9	Grassland
1305	18.3	1.8	BD	0.02	3.5	6.5	Loam	10.3	Grassland

Texture was determined according to the USDA referential. C_org_: organic carbon content; N: total nitrogen content; P: available phosphorous; K: total potassium content. *, **, ***: significant differences between Land Use type for edaphic parameters: P<0.05; P<0.01; P<0.001; respectively (Kruskal-Wallis non parametric test). BD: below the detection threshold.

**Figure 5:**
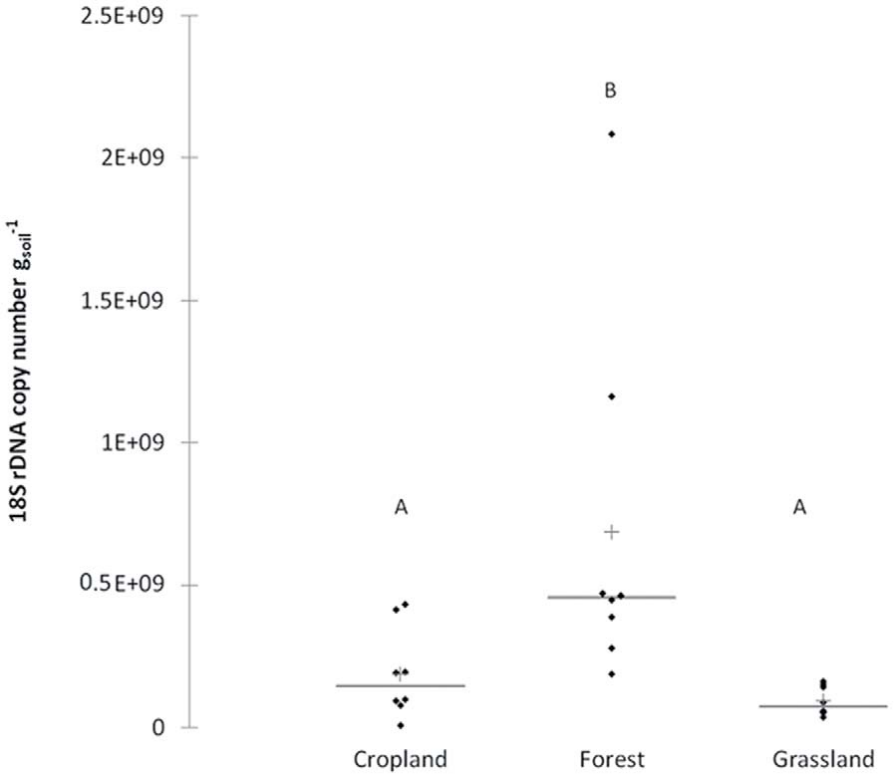
Variations of 18S rRNA gene copy number with land use type for 24 soil samples. Each dot represents the average 18S rRNA gene copy number for one soil sample. Cross and horizontal bars represent the mean and median 18S rRNA gene copy number for the land use type, respectively. Superscript letters indicate significant differences in copy numbers between land use (*P*<0.05).

Fungal abundance ranged from 6.9 10^6^ to 2.1 10^9^ copies of 18S rRNA gene .g^−1^ of soil (Figure 5, raw data are provided in Table S7) and was significantly correlated with soil physico-chemical properties (Table 3). Fine-textured soils exhibited a higher fungal abundance than coarse-textured soils, fungal abundance being negatively correlated with fine sand content. This observation is in agreement with the large number of bacterial and fungal organisms, as well as the greater microbial biomass, generally observed in silt or clay soils [45]–[47]. Fine-textured soils provide a more favorable habitat for microbial growth than coarse soils, offering better protection from desiccation, gas diffusion, toxic exogenous compounds and predation by protozoa [48]. Furthermore, the availability of carbon and nitrogen nutrient resources for indigenous microbes is generally higher in fine-textured soils due to better protection of the organic matter [49]. In addition, fungal abundance was significantly and positively correlated with C_org_ content and C∶N ratio. This confirmed the major contribution not only of the availability of organic matter but also of its biochemical quality, the C∶N ratio reflecting the recalcitrance of soil organic matter to microbial degradation, in agreement with other studies on soil fungal biology [9], [19], [46], [50], [51]. This would accord with the trophic niche differentiation between bacteria and fungi proposed by de Boer et al [9]. According to these authors, fungi preferentially decompose complex organic matter (cellulose, lignin) and interact with soil bacteria through co-metabolism of the fungal exudates. Under these conditions, an accumulation of complex organic matter would promote fungal development and increase fungal abundance (the case in this study as C_org_ and the C∶N ratio are correlated; r = 0.54; *P*<0.05). Nevertheless, this hypothesis needs to be tested by sampling on a larger scale under a broader range of C_org_ and C∶N ratio conditions. It has been demonstrated that other soil parameters may also be involved in determining the abundance of the soil fungal community: *e.g.* pH [52], P content [19] or N content [16], [53], [54]. These observations were not confirmed in this study. This could be i) because the gradients of pH, P content and N content between the different ecosystems were too small, thus preventing the observation of significant trends in the response of soil fungal abundance to these parameters, or ii) because different fungal phyla may respond differently to these parameters (*e.g.* Phosphorous, in Lauber et al [19]), or iii) because these parameters interacted with each other to influence the abundance of the soil fungal community.

**Table 3:** Pearson's correlation coefficients of 18S copy number and physico-chemical parameters.

Variable	18S (copies g^−1^ of dry soil)
Fine Sand (g kg^−1^)	−0.41*
C_org_ (g kg^−1^)	0.49*
C∶N	0.54*
N (g kg^−1^)	0.36
Clay (g kg^−1^)	0.32
Fine Loam (g kg^−1^)	0.29
pH_water_	0.20
CaCO_3_ (g kg^−1^)	0.10
Coarse Loam (g kg^−1^)	0.02
P (g kg^−1^)	−0.22
K (%)	−0.22
Coarse Sand (g kg^−1^)	−0.30

*: P<0.05.

Figure 5 shows the number of 18S rRNA gene copies for different land-use types, *i.e.* forests, croplands and grasslands. Forest sites contained a significantly (*P*<0.05) higher average number of copies of 18S rRNA gene.g^−1^ of soil (6.9 10^8^ copies.g^−1^ of soil) than grassland and cropland sites, which did not differ significantly from each other, (9.5 10^7^ and 1.9 10^8^ copies.g^−1^ of soil, respectively). This difference was significant despite the greater variability in the number of 18S rRNA gene.g^−1^ of soil observed in forest sites, as compared with grassland and cropland sites, (coefficients of variation: 87%, 78% and 50% for forest sites, cropland sites and grassland sites, respectively). These results are in agreement with those reported in the literature [17], [27]. The greater abundance of fungi in forest sites, compared to croplands and grasslands, may be related to their higher C∶N ratio and higher C_org_ content, showing that interactions between soil characteristics and vegetation type may also affect soil fungal abundance [55]. In addition, the high variability of soil fungal abundance in forests may partly be due to variations in the response of fungal groups to the C∶N ratio (positive or negative correlation, [19]) and the multiple symbiotic associations between plants and soil fungi. On the other hand, the higher number of copies of 18S rRNA gene g^−1^ of soil observed in croplands, as compared to grasslands, might be due to the higher P content of croplands. Indeed, significant differences in P content were observed between these land-use types and P content has been shown to influence the abundance of fungal populations in soil [19]. The identification of numerous edaphic variables influencing soil fungal abundance, in agreement with the literature, demonstrates that our tool is valid and operational for studying the determinism of fungal abundance in soil.

### Conclusion

In conclusion, the FR1 / FF390 primer set should facilitate the quantification of fungi in soils. Our results provide technical and ecological validation of combining use of the FR1 / FF390 primer set with a real-time Q-PCR approach and SYBRGreen® technology, to estimate fungal abundance in soils. The FR1 / FF390 primer set is the best consensus between fungi-specificity, coverage and a short amplicon among the different primer sets tested *in silico*, provides estimates of fungal abundance which are at least as accurate and reproducible as other primer sets in the literature, and avoids the reproducibility limitations associated with length polymorphism associated with the ITS region. Nevertheless, as with other primer sets, the true fungal abundance may be slightly underestimated because of incomplete coverage of the *Fungi* kingdom. However, this underestimation should remain weakly significant because it is related mainly to basal fungal lineages which constitute a small proportion of the fungal taxa currently referenced in the fungal databases. The major fungal taxa are equally and almost completely covered. In addition, this primer set is suitable for studying the determinism of soil fungal abundance. The analysis of 24 soil samples showed that the main determinants of soil fungal abundance, in this study, were soil type and land use. All these observations demonstrate that our tool is valid and operational for studying fungal abundance determinism in soil. However, to fully identify such determinism, it now needs to be applied to a large-scale soil sampling scheme, *e.g.* the European soil survey (particularly in France, UK, Holland and Germany [2]).

## Materials and Methods

### Soil Sampling

Soil samples were provided by the Soil Genetic Resource Center (platform GenoSol, http://www.dijon.inra.fr/plateforme_ genosol, [56]) and were obtained from the soil storage facility of the RMQS (“Réseau de Mesures de la Qualité des Sols” = French Monitoring Network for Soil Quality), which is a soil sampling network based on a 16×16 km systematic grid covering the whole of France [57]. The RMQS consists of 2,195 monitoring sites which have been geo-positioned. Soil profile, site environment, climatic factors, vegetation and land-use were described. Soil samples were air dried under controlled conditions (30°C, hygrometry) and then conserved at −40°C prior to DNA extraction. Five of these soils, contrasting in terms of texture, C and N content, pH and land-use were used to validate primer specificity under real-time Q-PCR conditions (soil characteristics reported in Table 1) and to test the reproducibility of the method. Separately, 24 other independent soil samples were analyzed to test the sensitivity and ecological potential of this tool by ranking the influence of soil properties and land-use practices on soil fungal abundance (physico-chemical characteristics provided in Table 2).

Several physico-chemical parameters were measured on each soil *i.e.*, particle-size distribution, pH water, organic carbon content (C_org_), N, C∶N ratio, soluble P contents, CaCO_3_, CEC and exchangeable cations (Ca, Mg). Physical and chemical analyses were performed by the Soil Analysis Laboratory of INRA (Arras, France) which is accredited for soil and sludge analysis and recognized by the French Ministry of Agriculture. Land-use was recorded according to the coarse level of the CORINE Land Cover classification (IFEN, http://www.ifen.fr) and consisted, for this study, of a rough descriptive classification into three classes: forest, crop systems and grassland.

### DNA Extraction and Purification from Soil Samples

For each soil sample, the equivalent of 1.5 g of dry soil was used for DNA extraction, following the procedure described in Ranjard et al. [58] and optimized by platform GenoSol (INRA, France, [56]). Briefly, extraction buffer (100 mM Tris pH 8.0, 100 mM EDTA pH 8.0, 100 mM NaCl and 2% (w/v) SDS) was added to the sample in the proportion 3∶1 (v/w), with two grams of glass beads (106 µm diameter) and eight glass beads (2 mm diameter) in a bead-beater tube. All beads were acid washed and sterilized. The samples were homogenized for 30 s at 1600 rpm in a mini bead-beater cell disruptor (Mikro-dismembrator, S. B. Braun Biotech International), incubated for 30 min at 70°C in a water bath and centrifuged for 5 min at 7000 g and room temperature. The supernatant was collected, incubated on ice with 1/10 volume of 3 M potassium acetate (pH 5.5) and centrifuged for 5 min at 14000 g. DNA was precipitated with one volume of ice-cold isopropanol and centrifuged for 30 min at 13000 rpm. The DNA pellet was washed with ice-cold 70% ethanol and dissolved in 100 µl of ultra pure water. The amount of crude DNA was determined by electrophoretic migration on a 1% agarose gel. The resulting DNA amount was reported to the amount of dry soil to determine the concentration of DNA in ng g^−1^ of dry soil.

For purification, aliquots (100 µL) of crude DNA extracts were loaded onto PVPP (polyvinyl polypyrrolidone) minicolumns (BIORAD, Marne la Coquette, France) and centrifuged for 4 min at 1000 g and 10°C. This step was repeated if the eluate was opaque. The eluate was then collected and purified for residual impurities using the Geneclean Turbo kit as recommended by the manufacturer (Q Biogene®, France).

### Primer Set FR1 / FF390

The primer set FR1 (5′-AICCATTCAATCGGTAIT-3′) / FF390 (5′-CGATAACGAACGAGACCT-3′) was developed by Vainio and Hantula [29]. This primer set is located at the end of the SSU 18S rRNA gene, near the ITS1 region, and has been shown to be appropriate for DGGE analysis of wood-inhabiting fungal communities. PCR amplification with this primer set produces PCR fragments of *ca.* 390 bp, suitable for real-time quantitative PCR, with only slight variations due to small length polymorphism.

#### *In Silico* Analyses

Dedicated C and Python programs (R. Christen, personal communication) were developed to analyze the different primer sets (primer sets detailed in Data S1, Sheet 01_Primers_list”). We used these programs to search large DNA sequence databases (such as 1 million SSU rRNA sequences) for the presence of primers, including degeneracies as coded by the IUPAC rules and also additional mismatches in order to test the primer improvement. The sequences investigated were Silva [59], direct extraction of every SSU rRNA sequence from EMBL using acnuc [60] and a dedicated reference database of 18S eukaryotic sequences which have been thoroughly analyzed and annotated (http://keydnatools.com, [61]).

First, a series of 18S sequences of fungi containing most of the 65 unique primers was retrieved from the Silva database. Primers were aligned in order to precise their locations (column “position”; Data S1, Sheet “02_Primer_selection”). Next, analyses using the databases described above allowed the evaluation of each primer individually for their yield for fungi with 0, 1, 2 and 3 mismatches and to select a subset of “good” primers. The selection criterion was the ratio between the number of sequences matched at k = 2 and k = 0. This ratio measures whether the primer detected significantly more fungal sequences with two mismatches than with no mismatch. A well designed primer was therefore a primer that has a small ratio k2/k0 (threshold set at 1.2), because it cannot be improved using more degeneracies. Primers with large ratio k2/k0 were discarded from the following analyses. A good primer is a primer that binds with a high percentage to every fungal clade but to a much lower extend to non fungal clades.

Second, the selected primers were combined into 33 primer sets. The relevant primer set was selected according to the length of the amplicon produced, its specificity and coverage for *Fungi*. A subset was derived from theses 33 primer sets according to the length of the amplicon produced that should be short [14] to enhance the accuracy and the reproducibility of the method (Data S1, Sheet “03_Selected_sets”). The threshold was determined by the length of the amplicon produced by the primer set nu-SSU-0817/nu-SSU-1196, a primer set previously used in combination with real-time Q-PCR: 384 bp. This resulted in the selection of a subset of 23 primer sets that were tested for their specificity and coverage for *Fungi* with exact match (Data S1, Sheet “04.1_Sets_evaluation”). This was performed on the Silva Reference sequence database (release 102) to check if the primer sets would not match bacterial or archaeal groups (495,824 Reference Sequences for the SSU genes) because these are well checked, unlike Eukaryotic sequences. Our own well-annotated database of 21,080 eukaryotic SSU rRNA gene sequences was used to check that no fungal group would be missed and also to see if other eukaryotic phyla could be detected by the different primer sets. Note that some sequences which were very short were not used. The yield for each primer set was retrieved and primers were compared to a theoretical optimal primer set (matching only fungal sequences and every fungal sequence) to determine which primer sets would be the more specific and would have the best coverage of *Fungi*. This was done through an ascendant hierarchical classification on the pearson's correlation coefficient similarity matrix based on centred and scaled data (raw data provided in Data S1, Sheet “04.2_Sets_evaluation_HAC”). The best primer sets that clustered with the theoretical optimal primer set were: nu-SSU-0817/nu-SSU-1196; FF390/FR1 ; nssu897R/nu-SSU-1196 and nssu1088R/SR2.

Among these four primer sets, the specificity for *Fungi* was checked in details to determine which one is best for the real-time Q-PCR approach. Different numbers of mismatches (0, 1, 2, 3) were allowed in the analysis to see if the primer set sequences to be used in real-time Q-PCR could be improved : a primer set can be improved if inserting mismatches significantly increases the hit frequency in the targeted phylum without increasing the hit frequency of non-targeted phyla.

#### Real-Time Q-PCR Conditions

For each soil DNA extract, the real-time Q-PCR products were amplified on an ABI PRISM 7900HT (Applied Biosystems, France) using SYBRGreen® as detection system in a reaction mixture of 20 µl containing 1.25 µM of each primer, 500 ng of T4 gene 32 protein (Appligen, France), 10 µl of SYBR Green PCR master mix, including HotStar Taq™ DNA polymerase, QuantiTec SYBR Green PCR Buffer, dNTP mix with dUTP, SYBR Green I, ROX and 5 mM MgCl2 (QuantiTec, SYBR Green PCR Kit, QIAGEN, France), 2 µl of template DNA, and DNAse – RNAse-free water to complete the final 20 µl volume.

The real-time Q-PCR conditions consisted of an initial step of 600 s at 95°C for enzyme activation, a second step corresponding to the PCR cycle (40 cycles) with 15 s at 95°C, 30 s at 50°C for hybridization, and an elongation step of 60 s at 70°C. Data were acquired at the end of this elongation step. A final step was added to obtain a specific denaturation curve from 70°C to 95°C with increments of 0.2°C s^−1^. Purity of the amplified products was checked by observation of a single melting peak and the presence of a single band of the expected length on 2% agarose gel stained with ethidium bromide. Real-time Q-PCR products obtained from DNA from a pure culture of *Fusarium oxysporum* 47 (INRA Dijon fungal collection) were cloned in a plasmid (pGEM-T Easy Vector System, Promega, France) and used as standard for the real-time Q-PCR assay after quantification with a Biophotometer Plus (Eppendorf, Germany). As purified soil DNA extracts may still contain PCR inhibitors, serial dilutions of the DNA templates (obtained from the 5 soils used to validate FR1 / FF390 primer set specificity) were used to determine the amount of DNA to be used in the real-time Q-PCR assay. The quantities of purified DNA used per well were 10 ng, 5 ng, 2.5 ng, 1 ng, and 0.5 ng.

#### Clone Library Construction and Sequencing

The PCR products obtained from the five soils used to set up the real-time Q-PCR conditions (template quantity: 2.5 ng) were cloned into the pGEM-T Easy Vector System (Promega, France) according to the manufacturer's instructions. Eighty-nine clones, distributed across the 5 soil samples (number of clones per sample: 7 to 38), were isolated. The DNA of each clone was extracted by “heat/cold” shocks. The plasmid inserts from each clone were amplified using Sp6 and T7 primers. The amplicons were run in 1.5% w/v agarose gel to determine the length of the insert. The inserts were sequenced using the SP6 primer (Cogenics, Meylan, France) and the resulting sequences were deposited in GenBank under the accession numbers referenced in Table S2.

#### Sequence Identification

Clone sequences were cleaned of plasmid sequence fragments (VecScreen, GENBANK) and affiliated using NCBI-Blast [62].

The distribution of clones sequences in different fungal groups was evaluated by aligning the sequences against reference sequences (Table S3) using seaview [63] and the maximum parsimony tree was computed using Phylowin [63] and visualized with the Dendroscope program [64].

### Statistical Analysis

The number of 18S rRNA copies ng^−1^ of DNA derived the real-time Q-PCR measurements were converted to a number of 18S rRNA copies g^−1^ of dry soil to allow the comparison between soil samples. A Kruskal-Wallis test was applied to check for significant differences in 18S rRNA gene copy number between the soils. Land-use types were compared with each other by multiple pair comparison. Correlations between soil physico-chemical characteristics and fungal 18S rRNA gene copy number were investigated by applying Pearson's correlation coefficient to the raw data. The significance level was set at the 5% probability level.

## Supporting Information

Figure S1 **Amplicon length distribution for the 33 primer sets tested in the** ***in silico*** **analysis.** Red dashed line represents the amplicon length threshold set by the primer set nu-SSU-0817/nu-SSU-1196. A primer set was selected for the next steps if the in silico analysis if its amplicon length was below the threshold limit.
(TIF)

Figure S2 **Hierarchical ascendant classification of the primer sets.** Dotted line: significance threshold at the 5% probability level. Clusters above the threshold limit are significant.
(TIF)

Figure S3 **Distribution of clones obtained by the cloning-sequencing approach in the different fungal phyla without introducing reference sequences.** Numbers on dendrogram branches are bootstrap values. Colors correspond to the phyla to which clones were affiliated as documented in Table S2.
(TIF)

Table S1 **Detailed hit frequencies (%) of the in silico analysis of FR1/FF390 and nu-SSU-0817/nu-SSU-1196 primer sets for Bacteria, Archaea, Eukaryota, eukaryotic phyla and fungal phyla.** The analysis allowed k mismatches, k ranging from 0 (original primer set sequences) to 3 (test of primer set sequences improvement).
(DOC)

Table S2 **Clone sequences affiliation, sequence length and accession numbers in GENBANK database.** na: not available.
(DOC)

Table S3 **Affiliation and accession numbers of reference sequences from GENBANK database.**
(DOC)

Table S4 **Glomeromycota amplification on pure culture DNA extracts by real time Q-PCR in combination with FR1/FF390 primer set.** NAN: Not A Number. The concentration of DNA extracts from pure cultures of *Glomus sp.* was not determined because very small volumes were available. This precluded having accurate estimates of the number of 18S rRNA gene copies in *Glomus sp.* extracts in this test. Nevertheless, the aim of this test was only to check if *Glomus sp.* DNA was amplified by the primer set FR1/FF390 in real-time Q-PCR conditions, which was the case. BD: lower than detection threshold.
(DOC)

Table S5 **Glomeromycota amplification on** ***Medicago truncatula*** **rhizosphere DNA extracts by real time Q-PCR in combination with FR1/FF390 primer set.**
(DOC)

Table S6 **Real-Time Q-PCR amplification results for the 5 soil samples used to test the specificity for fungi of FR1/FF390 primer set and to set up the template quantity in the real-time Q-PCR assay.** NAN: Not A Number.
(DOC)

Table S7 **Real-Time Q-PCR amplification results for the 24 soil samples used for the ecological validation of real-time Q PCR in combination with FR1/FF390 primer set.** NAN: Not A Number.
(DOC)

Data S1 ***In silico*** **analysis of literature primers and primer set selection. Sheet 01_Primers_List. List of the primers tested in the in silico analysis. Sheet 02_Primer_selection. Individual evaluation of each primer and primer selection results.** Each primer was evaluated individually for its yield for fungi with 0, 1, 2 and 3 mismatches. A subset of “good” primers was selected according to the ratio between the number of sequences matched at k = 2 and k = 0 which measured whether the primer detected significantly more fungal sequences with two mismatches than with no mismatch. A well designed primer was therefore a primer that has a small ratio k2/k0 (threshold set at 1.2), because it cannot be improved using more degeneracies. Primers with large ratio k2/k0 were discarded from the following analyses. A good primer is a primer that binds with a high percentage to every fungal clade but to a much lower extend to non fungal clades. **Sheet 03_Selected sets. Primer sets evaluation for the length of the amplicon produced by PCR.** A subset of primer sets was selected according to the length of the amplicon produced. The selection criterion was a short amplicon, shorter than the threshold limit determined by the length of the amplicon produced by primer set nu-SSU-0817–nu-SSU-1196. **Sheet 04.1_Sets_evaluation. Fungal specificity and coverage evaluation of each primer set with exact match.** Data presented are the hit frequency (%) of each primer set for each phyla. **Sheet 04.2_Sets_evaluation_HAC. Raw data for hierarchical ascendant classification analysis of the primer sets. Data presented are a number of matched sequences. The first raw indicates the names of the different phyla matched by the primer sets.** Number into brackets represent the total number of sequences per phyla. The results of the hierarchical ascendant classification analysis are presented in Figure S2.
(XLS)
