# Supplementary figures and images for: Validation and Application of a PCR Primer Set to Quantify Fungal Communities in the Soil Environment by Real-Time Quantitative PCR
Source: PLoS One. 2011 Sep 8;6(9):e24166. doi: 10.1371/journal.pone.0024166 (PMC3169588; doi:10.1371/journal.pone.0024166)

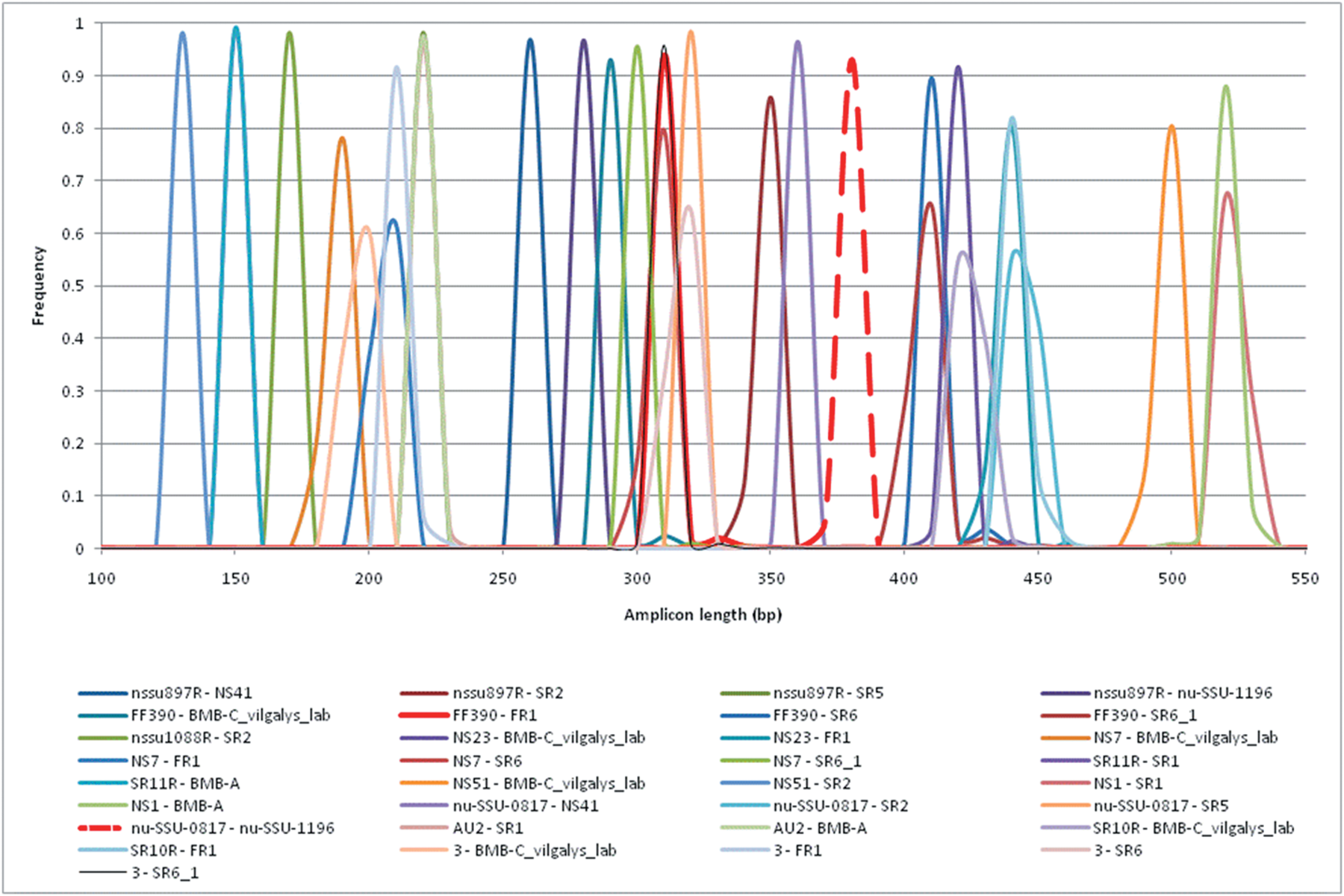

Supplement: Figure S1 — Amplicon length distribution for the 33 primer sets tested in the in silico analysis. Red dashed line represents the amplicon length threshold set by the primer set nu-SSU-0817/nu-SSU-1196. A primer set was selected for the next steps if the in silico analysis if its amplicon length was below the threshold limit. (TIF) [file pone.0024166.s001.tif]

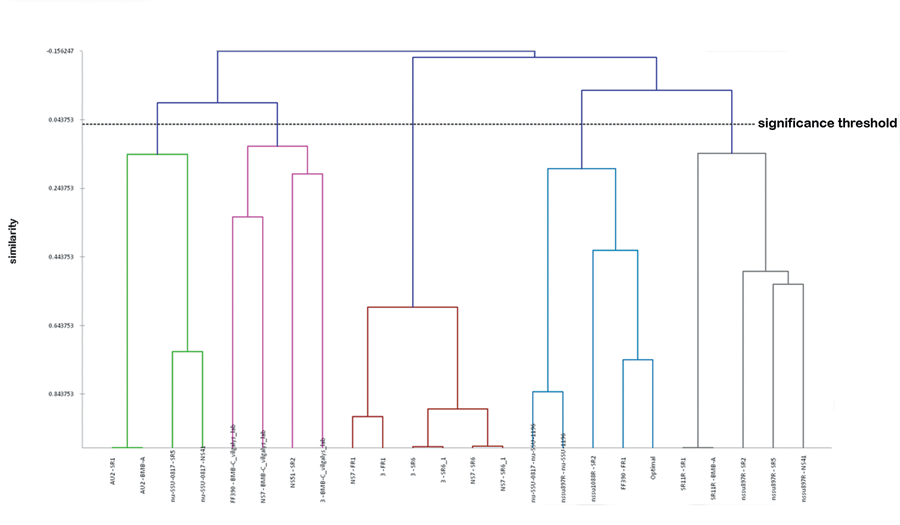

Supplement: Figure S2 — Hierarchical ascendant classification of the primer sets. Dotted line: significance threshold at the 5% probability level. Clusters above the threshold limit are significant. (TIF) [file pone.0024166.s002.tif]

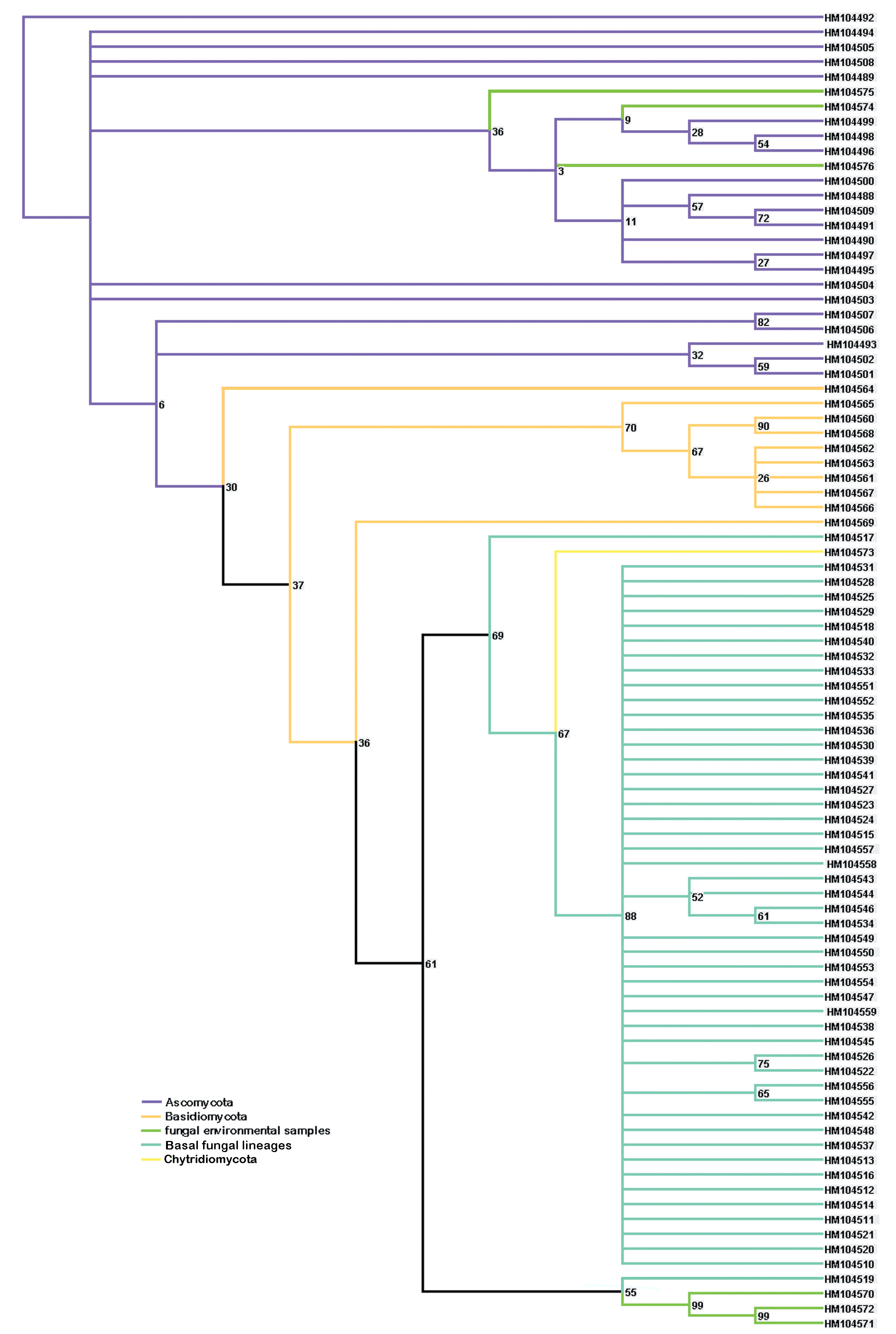

Supplement: Figure S3 — Distribution of clones obtained by the cloning-sequencing approach in the different fungal phyla without introducing reference sequences. Numbers on dendrogram branches are bootstrap values. Colors correspond to the phyla to which clones were affiliated as documented in Table S2. (TIF) [file pone.0024166.s003.tif]
