# Supplementary material for: Validation and Application of a PCR Primer Set to Quantify Fungal Communities in the Soil Environment by Real-Time Quantitative PCR
Source: PLoS One. 2011 Sep 8;6(9):e24166. doi: 10.1371/journal.pone.0024166 (PMC3169588; doi:10.1371/journal.pone.0024166)
Supplement: Table S1 — Detailed hit frequencies (%) of the in silico analysis of FR1/FF390 and nu-SSU-0817/nu-SSU-1196 primer sets for Bacteria, Archaea, Eukaryota, eukaryotic phyla and fungal phyla. The analysis allowed k mismatches, k ranging from 0 (original primer set sequences) to 3 (test of primer set sequences improvement). (DOC) [file pone.0024166.s004.doc]

**Table S1. Detailed hit frequencies (%) of the in silico analysis of FR1/FF390 and nu-SSU-0817/nu-SSU-1196 primer sets for Bacteria, Archaea, Eukaryota, eukaryotic phyla and fungal phyla**.

The analysis allowed k mismatches, k ranging from 0 (original primer set sequences) to 3 (test of primer set sequences improvement).

|  | **nssu897R - nu-SSU-1196** | | | | **FF390 - FR1** | | | | **nssu1088R - SR2** | | | | **nu-SSU-0817 - nu-SSU-1196** | | | |
| --- | --- | --- | --- | --- | --- | --- | --- | --- | --- | --- | --- | --- | --- | --- | --- | --- |
| **k=** | **k0** | **k1** | **k2** | **k3** | **k0** | **k1** | **k2** | **k3** | **k0** | **k1** | **k2** | **k3** | **k0** | **k1** | **k2** | **k3** |
| Archaea (23150) | ∅ | ∅ | ∅ | ∅ | ∅ | ∅ | ∅ | ∅ | ∅ | ∅ | ∅ | ∅ | ∅ | ∅ | ∅ | ∅ |
| Bacteria (472674) | ∅ | ∅ | ∅ | ∅ | ∅ | ∅ | ∅ | ∅ | ∅ | ∅ | ∅ | ∅ | ∅ | ∅ | ∅ | ∅ |
| Eukaryota (21080) | 12.8% | 32.5% | 49.7% | 61.9% | 10.3% | 18.2% | 44.1% | 55.9% | 8.4% | 14.1% | 30.9% | 43.5% | 11.4% | 22.0% | 36.7% | 51.7% |
| Incertae sedis Eukaryota Indien CladeG (1) | ∅ | ∅ | ∅ | 100.0% | ∅ | ∅ | ∅ | ∅ | ∅ | ∅ | ∅ | ∅ | ∅ | ∅ | ∅ | ∅ |
| Incertae sedis Eukaryota Indien CladeH (1) | ∅ | ∅ | 100.0% | 100.0% | ∅ | ∅ | ∅ | ∅ | ∅ | ∅ | ∅ | ∅ | ∅ | ∅ | ∅ | ∅ |
| Breviatea (11) | ∅ | ∅ | 72.7% | 90.9% | ∅ | ∅ | ∅ | ∅ | ∅ | 72.7% | 72.7% | 100.0% | ∅ | ∅ | 54.5% | 72.7% |
| Collodictyonidae (1) | ∅ | ∅ | 100.0% | 100.0% | ∅ | ∅ | ∅ | ∅ | ∅ | 100.0% | 100.0% | 100.0% | ∅ | ∅ | 100.0% | 100.0% |
| Alveolata (3621) | 1.8% | 50.2% | 72.4% | 85.0% | ∅ | 0.1% | 0.7% | 7.2% | 2.5% | 9.3% | 46.7% | 72.7% | ∅ | 20.6% | 45.3% | 64.2% |
| Apusomonadidae (14) | ∅ | 21.4% | 92.9% | 92.9% | ∅ | 7.1% | 50.0% | 85.7% | ∅ | 7.1% | 64.3% | 100.0% | ∅ | ∅ | 7.1% | 28.6% |
| Conosa (217) | 0.9% | 4.6% | 9.7% | 18.9% | ∅ | 1.8% | 3.2% | 17.5% | ∅ | ∅ | 18.9% | 27.2% | ∅ | 1.4% | 3.7% | 7.4% |
| Lobosa (246) | 2.4% | 28.9% | 60.6% | 74.8% | ∅ | 0.8% | 20.3% | 42.3% | ∅ | 13.0% | 23.6% | 35.8% | ∅ | 6.5% | 54.1% | 69.9% |
| Hilomonadea (16) | 6.3% | 6.3% | 62.5% | 62.5% | ∅ | ∅ | ∅ | 25.0% | ∅ | ∅ | 62.5% | 62.5% | ∅ | 6.3% | 56.3% | 56.3% |
| Amoebozoa incertae sedis (2) | ∅ | ∅ | ∅ | 50.0% | ∅ | ∅ | ∅ | ∅ | ∅ | ∅ | ∅ | ∅ | ∅ | ∅ | ∅ | ∅ |
| Chlorophyta (770) | 0.1% | 21.2% | 90.4% | 93.6% | ∅ | ∅ | 11.4% | 14.2% | 3.1% | 14.9% | 74.9% | 90.0% | ∅ | ∅ | 0.9% | 56.0% |
| Chlorarachniophyta nucleomorph (13) | ∅ | ∅ | ∅ | 15.4% | ∅ | ∅ | ∅ | ∅ | ∅ | ∅ | 76.9% | 100.0% | ∅ | ∅ | ∅ | ∅ |
| Cryptophyta nucleomorph (92) | ∅ | 1.1% | 17.4% | 67.4% | ∅ | ∅ | 1.1% | 53.3% | ∅ | ∅ | 1.1% | 40.2% | ∅ | 1.1% | 17.4% | 68.5% |
| Rhodophyta (823) | 0.2% | 2.3% | 36.6% | 65.5% | ∅ | ∅ | 14.1% | 86.4% | ∅ | 0.4% | 6.7% | 21.9% | ∅ | 1.1% | 35.4% | 63.4% |
| Streptophyta (512) | 7.0% | 82.2% | 93.8% | 96.5% | ∅ | 0.4% | 55.3% | 88.5% | 0.4% | 21.1% | 66.4% | 92.8% | ∅ | ∅ | 2.5% | 60.2% |
| Glaucocystophyta (5) | ∅ | ∅ | 100.0% | 100.0% | ∅ | ∅ | 100.0% | 100.0% | ∅ | ∅ | ∅ | ∅ | ∅ | ∅ | 100.0% | 100.0% |
| Palmophyllales (3) | ∅ | ∅ | 100.0% | 100.0% | ∅ | ∅ | ∅ | ∅ | ∅ | ∅ | ∅ | 100.0% | ∅ | ∅ | ∅ | 100.0% |
| Haptophyta (155) | ∅ | 25.2% | 95.5% | 96.1% | ∅ | 0.6% | 2.6% | 94.2% | ∅ | ∅ | 22.6% | 27.1% | ∅ | 85.2% | 91.0% | 92.9% |
| Katablepharidophyta (13) | ∅ | 76.9% | 92.3% | 92.3% | ∅ | ∅ | 38.5% | 92.3% | ∅ | ∅ | ∅ | 92.3% | ∅ | 76.9% | 92.3% | 92.3% |
| Malawimonadidae (2) | ∅ | 50.0% | 100.0% | 100.0% | ∅ | ∅ | ∅ | 100.0% | ∅ | ∅ | ∅ | ∅ | ∅ | ∅ | 100.0% | 100.0% |
| Discoba (770) | ∅ | 0.8% | 1.4% | 2.7% | ∅ | ∅ | ∅ | 0.5% | ∅ | ∅ | 0.9% | 1.0% | ∅ | ∅ | ∅ | ∅ |
| Metamonada (341) | 0.6% | 6.5% | 10.9% | 14.1% | ∅ | 0.3% | 0.3% | 10.6% | ∅ | ∅ | 3.5% | 20.5% | 1.2% | 7.0% | 10.6% | 10.6% |
| Centroheliozoa (53) | 24.5% | 90.6% | 96.2% | 100.0% | ∅ | ∅ | 18.9% | 20.8% | ∅ | ∅ | ∅ | ∅ | ∅ | 83.0% | 94.3% | 98.1% |
| Cryptophyta (133) | 10.5% | 94.7% | 98.5% | 100.0% | ∅ | 2.3% | 78.2% | 84.2% | ∅ | ∅ | 0.8% | 90.2% | 8.3% | 90.2% | 97.0% | 99.2% |
| Cercozoa (596) | ∅ | 4.2% | 54.0% | 76.0% | ∅ | 34.4% | 46.8% | 54.0% | ∅ | 0.7% | 18.5% | 46.5% | ∅ | 1.2% | 51.0% | 73.0% |
| Radiolaria (116) | ∅ | 9.5% | 31.9% | 85.3% | ∅ | ∅ | 1.7% | 53.4% | 2.6% | 37.9% | 67.2% | 84.5% | ∅ | ∅ | 9.5% | 43.1% |
| Foraminifera (509) | ∅ | ∅ | ∅ | 0.2% | ∅ | ∅ | ∅ | ∅ | ∅ | ∅ | ∅ | ∅ | ∅ | ∅ | ∅ | ∅ |
| Stramenopiles (1715) | 1.5% | 40.1% | 88.1% | 95.4% | ∅ | 0.9% | 66.6% | 81.8% | 0.5% | 4.5% | 53.0% | 70.6% | ∅ | 16.5% | 72.0% | 84.0% |
| Picobiliphyta (14) | ∅ | 14.3% | 92.9% | 92.9% | ∅ | ∅ | ∅ | 7.1% | 0.0% | ∅ | 7.1% | 100.0% | ∅ | 14.3% | 100.0% | 100.0% |
| Telonemia (35) | ∅ | ∅ | 80.0% | 88.6% | ∅ | 2.9% | 48.6% | 65.7% | 0.0% | 94.3% | 94.3% | 94.3% | ∅ | ∅ | 85.7% | 88.6% |
| Uncertain Hacrobia (1) | ∅ | 100.0% | 100.0% | 100.0% | ∅ | ∅ | ∅ | ∅ | ∅ | ∅ | ∅ | 100.0% | ∅ | ∅ | ∅ | 100.0% |
| Choanoflagellida (127) | 65.4% | 85.8% | 88.2% | 88.2% | 0.8% | 46.5% | 52.0% | 52.8% | 4.7% | 17.3% | 53.5% | 70.9% | 66.1% | 85.8% | 86.6% | 86.6% |
| Mesomycetozoa (65) | 40.0% | 55.4% | 87.7% | 96.9% | 10.8% | 89.2% | 89.2% | 89.2% | ∅ | 3.1% | 63.1% | 73.8% | 10.8% | 43.1% | 86.2% | 95.4% |
| Metazoa (6769) | 1.6% | 7.7% | 12.2% | 31.2% | 0.3% | 12.2% | 63.5% | 74.4% | 0.2% | 1.7% | 2.9% | 5.7% | 0.6% | 6.5% | 9.6% | 23.6% |
| Opisthokonta incertae sedis (6) | ∅ | ∅ | 100.0% | 100.0% | ∅ | 100.0% | 100.0% | 100.0% | ∅ | 100.0% | 100.0% | 100.0% | ∅ | ∅ | 100.0% | 100.0% |
| Fungi (3308) | 69.8% | 81.9% | 86.1% | 88.7% | 64.5% | 80.2% | 81.9% | 83.0% | 49.4% | 62.3% | 67.1% | 76.5% | 68.0% | 80.4% | 85.6% | 87.8% |
| Ascomycota (1817) | 82.0% | 90.3% | 92.7% | 93.1% | 73.7% | 84.4% | 86.0% | 86.6% | 68.0% | 83.7% | 85.9% | 87.5% | 79.2% | 88.1% | 91.0% | 92.2% |
| Pezizomycotina (1232) | 81.3% | 89.4% | 91.8% | 92.3% | 71.0% | 82.2% | 83.4% | 83.6% | 65.7% | 83.2% | 85.2% | 86.1% | 77.4% | 88.3% | 91.4% | 92.0% |
| Saccharomycotina (549) | 84.2% | 92.5% | 94.7% | 95.1% | 78.9% | 88.9% | 91.6% | 93.3% | 75.8% | 85.4% | 87.4% | 90.7% | 83.1% | 87.4% | 90.3% | 92.7% |
| Taphrinomycotina (36) | 72.2% | 88.9% | 91.7% | 91.7% | 88.9% | 88.9% | 88.9% | 88.9% | 30.6% | 75.0% | 86.1% | 86.1% | 80.6% | 88.9% | 88.9% | 91.7% |
| Basal fungi (812) | 42.6% | 54.1% | 65.3% | 73.3% | 33.7% | 65.5% | 67.6% | 68.1% | 1.4% | 9.4% | 21.2% | 49.9% | 31.0% | 55.9% | 68.2% | 72.4% |
| Blastocladiomycota (19) | 78.9% | 94.7% | 94.7% | 100.0% | 31.6% | 89.5% | 94.7% | 94.7% | ∅ | ∅ | 68.4% | 89.5% | 84.2% | 100.0% | 100.0% | 100.0% |
| Chytridiomycota (148) | 44.6% | 61.5% | 64.9% | 65.5% | 15.5% | 57.4% | 57.4% | 58.1% | 2.7% | 37.2% | 58.1% | 60.8% | 38.5% | 60.1% | 65.5% | 66.2% |
| Entomophthoromycotina (59) | 10.2% | 16.9% | 54.2% | 78.0% | 72.9% | 76.3% | 83.1% | 86.4% | ∅ | ∅ | 13.6% | 52.5% | 10.2% | 15.3% | 55.9% | 81.4% |
| Glomeromycota (192) | 59.4% | 79.2% | 96.9% | 97.4% | 29.7% | 89.1% | 91.1% | 91.1% | ∅ | ∅ | 6.8% | 84.9% | 59.4% | 79.7% | 97.4% | 98.4% |
| Kickxellomycotina (69) | 21.7% | 26.1% | 34.8% | 97.1% | 72.5% | 87.0% | 87.0% | 87.0% | ∅ | 4.3% | 5.8% | 62.3% | 37.7% | 73.9% | 91.3% | 95.7% |
| LKM11-Rozella (36) | 2.8% | 25.0% | 83.3% | 97.2% | 47.2% | 72.2% | 72.2% | 72.2% | 16.7% | 38.9% | 72.2% | 91.7% | 2.8% | 19.4% | 50.0% | 75.0% |
| Microsporidiomycota (138) | ∅ | ∅ | ∅ | ∅ | ∅ | ∅ | ∅ | ∅ | ∅ | ∅ | ∅ | 2.2% | ∅ | ∅ | ∅ | ∅ |
| Mucoromycotina (142) | 90.1% | 95.8% | 95.8% | 95.8% | 52.1% | 86.6% | 90.8% | 91.5% | ∅ | 0.7% | 10.6% | 11.3% | 21.8% | 85.2% | 90.8% | 93.7% |
| Nephridiophagidae (1) | ∅ | 100.0% | 100.0% | 100.0% | ∅ | ∅ | ∅ | ∅ | ∅ | ∅ | 100.0% | 100.0% | ∅ | 100.0% | 100.0% | 100.0% |
| Zoopagomycotina (8) | 12.5% | 50.0% | 87.5% | 87.5% | 50.0% | 62.5% | 87.5% | 87.5% | 12.5% | 37.5% | 75.0% | 100.0% | 12.5% | 50.0% | 87.5% | 87.5% |
| Basidiomycota (679) | 69.5% | 92.6% | 93.5% | 95.4% | 76.7% | 86.5% | 88.2% | 90.9% | 57.0% | 68.5% | 71.6% | 78.8% | 82.2% | 89.1% | 91.8% | 94.4% |
| Agaricomycotina (465) | 65.4% | 95.3% | 95.7% | 96.8% | 82.8% | 92.0% | 93.1% | 94.6% | 80.2% | 91.2% | 95.3% | 96.6% | 86.7% | 93.3% | 94.6% | 95.9% |
| Entorrhizomycetes (4) | 75.0% | 75.0% | 75.0% | 75.0% | ∅ | 75.0% | 75.0% | 75.0% | ∅ | 75.0% | 75.0% | 75.0% | 75.0% | 75.0% | 75.0% | 75.0% |
| Pucciniomycotina (149) | 85.2% | 89.9% | 90.6% | 92.6% | 65.1% | 73.8% | 76.5% | 83.2% | ∅ | ∅ | 0.7% | 23.5% | 73.8% | 79.2% | 85.9% | 91.3% |
