# Supplementary material for: Validation and Application of a PCR Primer Set to Quantify Fungal Communities in the Soil Environment by Real-Time Quantitative PCR
Source: PLoS One. 2011 Sep 8;6(9):e24166. doi: 10.1371/journal.pone.0024166 (PMC3169588; doi:10.1371/journal.pone.0024166)
Supplement: Table S2 — Clone sequences affiliation, sequence length and accession numbers in GENBANK database. na: not available. (DOC) [file pone.0024166.s005.doc]

**Table S2. Clone sequences affiliation, sequence length and accession numbers in GENBANK database.**

na: not available.

| **Affiliation (inside Fungi Kingdom)** | | **Fragment length** | **Accession number** |
| --- | --- | --- | --- |
| Chytridiomycota | Chytridiomycetes | 353 | HM104573 |
| Dikarya | Ascomycota | 348 | HM104488 |
| Dikarya | Ascomycota | 350 | HM104489 |
| Dikarya | Ascomycota | 349 | HM104490 |
| Dikarya | Ascomycota | 348 | HM104491 |
| Dikarya | Ascomycota | 350 | HM104492 |
| Dikarya | Ascomycota | 349 | HM104493 |
| Dikarya | Ascomycota | 350 | HM104494 |
| Dikarya | Ascomycota | 348 | HM104495 |
| Dikarya | Ascomycota | 348 | HM104496 |
| Dikarya | Ascomycota | 348 | HM104497 |
| Dikarya | Ascomycota | 348 | HM104498 |
| Dikarya | Ascomycota | 348 | HM104499 |
| Dikarya | Ascomycota | 348 | HM104500 |
| Dikarya | Ascomycota | 349 | HM104501 |
| Dikarya | Ascomycota | 349 | HM104502 |
| Dikarya | Ascomycota | 350 | HM104503 |
| Dikarya | Ascomycota | 350 | HM104504 |
| Dikarya | Ascomycota | 352 | HM104505 |
| Dikarya | Ascomycota | 349 | HM104506 |
| Dikarya | Ascomycota | 349 | HM104507 |
| Dikarya | Ascomycota | 359 | HM104508 |
| Dikarya | Ascomycota | 348 | HM104509 |
| Dikarya | Basidiomycota | 352 | HM104560 |
| Dikarya | Basidiomycota | 351 | HM104561 |
| Dikarya | Basidiomycota | 340 | HM104562 |
| Dikarya | Basidiomycota | 340 | HM104563 |
| Dikarya | Basidiomycota | 351 | HM104564 |
| Dikarya | Basidiomycota | 352 | HM104565 |
| Dikarya | Basidiomycota | 351 | HM104566 |
| Dikarya | Basidiomycota | 351 | HM104567 |
| Dikarya | Basidiomycota | 352 | HM104568 |
| Dikarya | Basidiomycota | 351 | HM104569 |
| Fungal environmental samples | na | 355 | HM104570 |
| Fungal environmental samples | na | 354 | HM104571 |
| Fungal environmental samples | na | 354 | HM104572 |
| Fungal environmental samples | na | 348 | HM104574 |
| Fungal environmental samples | na | 348 | HM104575 |
| Fungal environmental samples | na | 348 | HM104576 |
| Fungi incertae sedis | Basal fungal lineages | 351 | HM104510 |
| Fungi incertae sedis | Basal fungal lineages | 351 | HM104511 |
| Fungi incertae sedis | Basal fungal lineages | 351 | HM104512 |
| Fungi incertae sedis | Basal fungal lineages | 351 | HM104513 |
| Fungi incertae sedis | Basal fungal lineages | 351 | HM104514 |
| Fungi incertae sedis | Basal fungal lineages | 344 | HM104515 |
| Fungi incertae sedis | Basal fungal lineages | 351 | HM104516 |
| Fungi incertae sedis | Basal fungal lineages | 351 | HM104517 |
| Fungi incertae sedis | Basal fungal lineages | 317 | HM104518 |
| Fungi incertae sedis | Basal fungal lineages | 360 | HM104519 |
| Fungi incertae sedis | Basal fungal lineages | 351 | HM104520 |
| Fungi incertae sedis | Basal fungal lineages | 354 | HM104521 |
| Fungi incertae sedis | Basal fungal lineages | 351 | HM104522 |
| Fungi incertae sedis | Basal fungal lineages | 351 | HM104523 |
| Fungi incertae sedis | Basal fungal lineages | 351 | HM104524 |
| Fungi incertae sedis | Basal fungal lineages | 351 | HM104525 |
| Fungi incertae sedis | Basal fungal lineages | 351 | HM104526 |
| Fungi incertae sedis | Basal fungal lineages | 351 | HM104527 |
| Fungi incertae sedis | Basal fungal lineages | 351 | HM104528 |
| Fungi incertae sedis | Basal fungal lineages | 351 | HM104529 |
| Fungi incertae sedis | Basal fungal lineages | 351 | HM104530 |
| Fungi incertae sedis | Basal fungal lineages | 351 | HM104531 |
| Fungi incertae sedis | Basal fungal lineages | 351 | HM104532 |
| Fungi incertae sedis | Basal fungal lineages | 351 | HM104533 |
| Fungi incertae sedis | Basal fungal lineages | 351 | HM104534 |
| Fungi incertae sedis | Basal fungal lineages | 351 | HM104535 |
| Fungi incertae sedis | Basal fungal lineages | 351 | HM104536 |
| Fungi incertae sedis | Basal fungal lineages | 351 | HM104537 |
| Fungi incertae sedis | Basal fungal lineages | 351 | HM104538 |
| Fungi incertae sedis | Basal fungal lineages | 351 | HM104539 |
| Fungi incertae sedis | Basal fungal lineages | 351 | HM104540 |
| Fungi incertae sedis | Basal fungal lineages | 351 | HM104541 |
| Fungi incertae sedis | Basal fungal lineages | 351 | HM104542 |
| Fungi incertae sedis | Basal fungal lineages | 355 | HM104543 |
| Fungi incertae sedis | Basal fungal lineages | 351 | HM104544 |
| Fungi incertae sedis | Basal fungal lineages | 351 | HM104545 |
| Fungi incertae sedis | Basal fungal lineages | 351 | HM104546 |
| Fungi incertae sedis | Basal fungal lineages | 351 | HM104547 |
| Fungi incertae sedis | Basal fungal lineages | 351 | HM104548 |
| Fungi incertae sedis | Basal fungal lineages | 351 | HM104549 |
| Fungi incertae sedis | Basal fungal lineages | 351 | HM104550 |
| Fungi incertae sedis | Basal fungal lineages | 351 | HM104551 |
| Fungi incertae sedis | Basal fungal lineages | 351 | HM104552 |
| Fungi incertae sedis | Basal fungal lineages | 351 | HM104553 |
| Fungi incertae sedis | Basal fungal lineages | 351 | HM104554 |
| Fungi incertae sedis | Basal fungal lineages | 351 | HM104555 |
| Fungi incertae sedis | Basal fungal lineages | 351 | HM104556 |
| Fungi incertae sedis | Basal fungal lineages | 351 | HM104557 |
| Fungi incertae sedis | Basal fungal lineages | 351 | HM104558 |
| Fungi incertae sedis | Basal fungal lineages | 351 | HM104559 |
