# Supplementary material for: Validation and Application of a PCR Primer Set to Quantify Fungal Communities in the Soil Environment by Real-Time Quantitative PCR
Source: PLoS One. 2011 Sep 8;6(9):e24166. doi: 10.1371/journal.pone.0024166 (PMC3169588; doi:10.1371/journal.pone.0024166)
Supplement: Table S3 — Affiliation and accession numbers of reference sequences from GENBANK database. (DOC) [file pone.0024166.s006.doc]

**Table S3. Affiliation and accession numbers of reference sequences from GENBANK database.**

| **Affiliation** | **name** |  | **Accession number** | **Abbreviation** |
| --- | --- | --- | --- | --- |
| Alveolata; Apicomplexa; Aconoidasida; Piroplasmida | *Piroplasmida sp. AJB-2006* |  | EF057099 | Piroplasmida_sp |
| Metazoa; Acanthocephala; Archiacanthocephala; | *Moniliformis moniliformis* |  | Z19562 | Moniliformis_monili |
| Metazoa; Ctenophora; Cyclocoela; Lobata | *Mnemiopsis leidyi* |  | L10826 | Mnemiopsis_leidyi |
| Metazoa; Porifera; Calcarea; Calcaronea | *Sycon ciliatum* |  | L10827 | Sycon_ciliatum |
| Metazoa; Porifera; Demospongiae; Tetractinomorpha | *Tetilla japonica* |  | D15067 | Tetilla_japonica |
| Nucleariidae; Nuclearia.; ; | *Nuclearia thermophila* |  | AB433328 | Nuclearia_thermophil |
| Viridiplantae; Chlorophyta; Trebouxiophyceae; Chlorellales | *Parachlorella kessleri* |  | X56105 | Parachlorella_kessle |
| Viridiplantae; Streptophyta; Embryophyta; Tracheophyta | *Arabidopsis thaliana* |  | X16077 | Arabidopsis_thaliana |
| Viridiplantae; Streptophyta; Embryophyta; Tracheophyta | *Lonchitis hirsuta* |  | U18632 | Lonchitis_hirsuta |
| Viridiplantae; Streptophyta; Embryophyta; Tracheophyta | *Pinus elliottii* |  | D38245 | Pinus_elliottii |
| Fungi; Blastocladiomycota; Blastocladiomycetes; Blastocladiales | *Blastocladiella emersonii* |  | AY635842 | Blastocladiella_emer |
| Fungi; Blastocladiomycota; Blastocladiomycetes; Blastocladiales | *Catenomyces sp. JEL342* |  | AY635830 | Catenomyces_sp._JEL |
| Fungi; Blastocladiomycota; Blastocladiomycetes; Blastocladiales | *Catenophlyctis sp. JEL298* |  | AY635822 | Catenophlyctis_sp._JE |
| Fungi; Blastocladiomycota; Blastocladiomycetes; Blastocladiales | *Microallomyces sp. CR74* |  | AY635840 | Microallomyces_sp._CR |
| Fungi; Chytridiomycota; Chytridiomycetes; | *Powellomyces variabilis* |  | AF164244 | Powellomyces_variabi |
| Fungi; Chytridiomycota; Chytridiomycetes; Chytridiales | *Chytriomyces angularis* |  | AF164253 | Chytriomyces_angular |
| Fungi; Chytridiomycota; Chytridiomycetes; Chytridiales | *Karlingiomyces sp. JEL93* |  | AF164278 | Karlingiomyces_sp._JE |
| Fungi; Chytridiomycota; Monoblepharidomycetes; | *Hyaloraphidium curvatum* |  | Y17504 | Hyaloraphidium_curva |
| Fungi; Chytridiomycota; Monoblepharidomycetes; | *Monoblepharis macrandra* |  | EF014369 | Monoblepharis_macran |
| Fungi; Chytridiomycota; Monoblepharidomycetes; Monoblepharidales | *Monoblepharis sp. UBC70-1* |  | AB016019 | Monoblepharis_sp._UBC |
| Fungi; Dikarya; Ascomycota; | *Calcarisporium arbuscula* |  | AY271796 | Calcarisporium_arbus |
| Fungi; Dikarya; Ascomycota; | *Lecophagus sp. ATCC 56071* |  | AY635836 | Lecophagus_sp._ATCC |
| Fungi; Dikarya; Ascomycota; | *Loramyces macrosporus* |  | DQ471005 | Loramyces_macrosporu |
| Fungi; Dikarya; Ascomycota; | *Microdochium nivale* |  | AF548077 | Microdochium_nivale |
| Fungi; Dikarya; Ascomycota; | *Nectria lugdunensis* |  | AY357278 | Nectria_lugdunensis |
| Fungi; Dikarya; Ascomycota; | *Sporothrix schenckii* |  | M85053 | Sporothrix_schenckii |
| Fungi; Dikarya; Ascomycota; | *Stachybotrys kampalensis* |  | AF548099 | Stachybotrys_kampale |
| Fungi; Dikarya; Ascomycota; Pezizomycotina | *Chaetomium elatum* |  | M83257 | Chaetomium_elatum |
| Fungi; Dikarya; Ascomycota; Pezizomycotina | *Eremascus albus* |  | M83258 | Eremascus_albus |
| Fungi; Dikarya; Ascomycota; Pezizomycotina | *Morchella elata* |  | L37537 | Morchella_elata |
| Fungi; Dikarya; Ascomycota; Pezizomycotina | *Peziza badia* |  | L37539 | Peziza_badia |
| Fungi; Dikarya; Ascomycota; Pezizomycotina | *Pleospora rudis* |  | U00975 | Pleospora_rudis |
| Fungi; Dikarya; Ascomycota; Pezizomycotina | *Porpidia crustulata* |  | L37735 | Porpidia_crustulata |
| Fungi; Dikarya; Ascomycota; Pezizomycotina | *Sclerotinia sclerotiorum* |  | X69850 | Sclerotinia_scleroti |
| Fungi; Dikarya; Ascomycota; Pezizomycotina | *Thermoascus crustaceus* |  | M83263 | Thermoascus_crustace |
| Fungi; Dikarya; Ascomycota; Saccharomycotina | *Lipomyces lipofer* |  | X69848 | Lipomyces_lipofer |
| Fungi; Dikarya; Ascomycota; Saccharomycotina | *Vanderwaltozyma polyspor* |  | X69845 | Vanderwaltozyma_poly |
| Fungi; Dikarya; Ascomycota; Taphrinomycotina | *Neolecta vitellina* |  | Z27393 | Neolecta_vitellina |
| Fungi; Dikarya; Ascomycota; Taphrinomycotina | *Schizosaccharomyces pombe* |  | X58056 | Schizosaccharomyces |
| Fungi; Dikarya; Ascomycota; Taphrinomycotina | *Taphrina populina* |  | D14165 | Taphrina_populina |
| Fungi; Dikarya; Basidiomycota; | *basidiomycete yeast sp. BG02-6-6-1-9* |  | AY520283 | basidiomycete_yeast |
| Fungi; Dikarya; Basidiomycota; | *Coniophora marmorata* |  | AM946632 | Coniophora_marmorata |
| Fungi; Dikarya; Basidiomycota; | *Cystofilobasidium bisporidii* |  | AB072225 | Cystofilobasidium_bi |
| Fungi; Dikarya; Basidiomycota; | *Lepiota procera* |  | L36659 | Lepiota_procera |
| Fungi; Dikarya; Basidiomycota; | *Mycena plumbea* |  | DQ457697 | Mycena_plumbea |
| Fungi; Dikarya; Basidiomycota; | *Russula exalbicans* |  | AY293156 | Russula_exalbicans |
| Fungi; Dikarya; Basidiomycota; | *Trichosporon porosum* |  | AB051045 | Trichosporon_porosum |
| Fungi; Dikarya; Basidiomycota; Agaricomycotina | *Agaricus bisporus* |  | L36658 | Agaricus_bisporus |
| Fungi; Dikarya; Basidiomycota; Agaricomycotina | *Boletus satanas* |  | M94337 | Boletus_satanas |
| Fungi; Dikarya; Basidiomycota; Agaricomycotina | *Filobasidium floriforme* |  | D13460 | Filobasidium_florifo |
| Fungi; Dikarya; Basidiomycota; Agaricomycotina | *Heterotextus alpinus* |  | L22259 | Heterotextus_alpinus |
| Fungi; Dikarya; Basidiomycota; Pucciniomycotina | *Endocronartium harknessii* |  | M94339 | Endocronartium_harkn |
| Fungi; Dikarya; Basidiomycota; Ustilaginomycotina | *Ustilago hordei* |  | U00973 | Ustilago_hordei |
| Fungi; Fungiincertaesedis; Basalfungallineages; | *Mortierella indohii* |  | EU688965 | Mortierella_indohii |
| Fungi; Fungiincertaesedis; Basalfungallineages; | *Ramicandelaber brevisporus* |  | AB287987 | Ramicandelaber_brevi |
| Fungi; Fungiincertaesedis; Basalfungallineages; Entomophthoromycotina | *Basidiobolus ranarum* |  | AY635841 | Basidiobolus_ranarum |
| Fungi; Fungiincertaesedis; Basalfungallineages; Mucoromycotina | *Cokeromyces recurvatus* |  | AY635843 | Cokeromyces_recurvat |
| Fungi; Fungiincertaesedis; Basalfungallineages; unclassifiedzygomycetes | *zygomycete sp. AM-2008a* |  | EU428766 | zygomycete_sp._AM-a |
| Fungi; Glomeromycota; Glomeromycetes; Diversisporales | *Diversispora sp. AS-2007a* |  | AM713427 | Diversispora_sp._AS-a |
| Fungi; Glomeromycota; Glomeromycetes; Diversisporales | *Scutellospora heterogama* |  | AY635832 | Scutellospora_hetero |
| Fungi; Glomeromycota; Glomeromycetes; Glomerales | *Glomus eburneum* |  | AM713430 | Glomus_eburneum |
| Fungi; Microsporidia; Microsporidiaincertaesedis; | *Microsporidium prosopium* |  | AF151529 | Microsporidium_proso |
| Fungi; Microsporidia; Nosematidae; | *Nosema vespula* |  | L31842 | Nosema_vespula |
| Fungi; Microsporidia; Pansporablastina; Thelohania. | *Thelohania disparis* |  | DQ272237 | Thelohania_disparis |
