# Supplementary material for: Validation and Application of a PCR Primer Set to Quantify Fungal Communities in the Soil Environment by Real-Time Quantitative PCR
Source: PLoS One. 2011 Sep 8;6(9):e24166. doi: 10.1371/journal.pone.0024166 (PMC3169588; doi:10.1371/journal.pone.0024166)
Supplement: Table S4 — Glomeromycota amplification on pure culture DNA extracts by real time Q-PCR in combination with FR1/FF390 primer set. NAN: Not A Number. The concentration of DNA extracts from pure cultures of Glomus sp. was not determined because very small volumes were available. This precluded having accurate estimates of the number of 18S rRNA gene copies in Glomus sp. extracts in this test. Nevertheless, the aim of this test was only to check if Glomus sp. DNA was amplified by the primer set FR1/FF390 in real-time Q-PCR conditions, which was the case. BD: lower than detection threshold. (DOC) [file pone.0024166.s007.doc]

**Table S4. Glomeromycota amplification on pure culture DNA extracts by real time Q-PCR in combination with FR1/FF390 primer set.**

| Sample Name | Template dilution | Template quantity (ng) | Task | Cт | Quantity |
| --- | --- | --- | --- | --- | --- |
| *Glomus versiforme* | 1 | NAN | Sample | 25.6 | 7916 |
| *Glomus versiforme* | 1 :10 | NAN | Sample | 25.6 | 7964 |
| *Glomus versiforme* | 1 :100 | NAN | Sample | 29.3 | 470 |
| *Glomus versiforme* | 1:1000 | NAN | Sample | 31.8 | BD |
| *Glomus clarum* | 1 | NAN | Sample | 27.7 | 1566 |
| *Glomus clarum* | 1 :10 | NAN | Sample | 30.4 | 209 |
| *Glomus clarum* | 1 :100 | NAN | Sample | 33.6 | BD |
| *Glomus clarum* | 1:1000 | NAN | Sample | 33.8 | BD |
| *Glomus claroideum* | 1 | NAN | Sample | 26.6 | 3728 |
| *Glomus claroideum* | 1 :10 | NAN | Sample | 28.5 | 858 |
| *Glomus claroideum* | 1 :100 | NAN | Sample | 29.4 | 439 |
| *Glomus claroideum* | 1:1000 | NAN | Sample | 32.5 | BD |
| *Glomus geosporum* | 1 | NAN | Sample | 22.0 | 117285 |
| *Glomus geosporum* | 1 :10 | NAN | Sample | 23.2 | 48315 |
| *Glomus geosporum* | 1 :100 | NAN | Sample | 26.0 | 5803 |
| *Glomus geosporum* | 1:1000 | NAN | Sample | 31.7 | BD |
| *Positive template* | 1 | 2 | Positive Template | 13.5 | 7.00E+07 |
| *Positive template* | 1 | 2 | Positive Template | 13.3 | 8.50E+07 |
| Standard | NAN | NAN | STANDARD | 13.3 | 3.28E+07 |
| Standard | NAN | NAN | STANDARD | 13.4 | 3.28E+07 |
| Standard | NAN | NAN | STANDARD | 13.4 | 3.28E+07 |
| Standard | NAN | NAN | STANDARD | 17.8 | 3.28E+06 |
| Standard | NAN | NAN | STANDARD | 17.7 | 3.28E+06 |
| Standard | NAN | NAN | STANDARD | 17.4 | 3.28E+06 |
| Standard | NAN | NAN | STANDARD | 23.0 | 3.28E+05 |
| Standard | NAN | NAN | STANDARD | 22.5 | 3.28E+05 |
| Standard | NAN | NAN | STANDARD | 23.2 | 3.28E+05 |
| Standard | NAN | NAN | STANDARD | 25.9 | 3.28E+03 |
| Standard | NAN | NAN | STANDARD | 25.4 | 3.28E+03 |
| Standard | NAN | NAN | STANDARD | 25.5 | 3.28E+03 |
| Standard | NAN | NAN | STANDARD | 29.4 | 3.28E+02 |
| Standard | NAN | NAN | STANDARD | 29.8 | 3.28E+02 |
| Standard | NAN | NAN | STANDARD | 30.2 | 3.28E+02 |
| Negative template | NAN | 0 | NTC | 33.9 |  |
| Negative template | NAN | 0 | NTC | 33.7 |  |

NAN: Not A Number.

The concentration of DNA extracts from pure cultures of *Glomus sp.* was not determined because very small volumes were available. This precluded having accurate estimates of the number of 18S rRNA gene copies in *Glomus sp.* extracts in this test. Nevertheless, the aim of this test was only to check if *Glomus sp.* DNA was amplified by the primer set FR1/FF390 in real-time Q-PCR conditions, which was the case. BD: lower than detection threshold
