# Supplementary material for: Validation and Application of a PCR Primer Set to Quantify Fungal Communities in the Soil Environment by Real-Time Quantitative PCR
Source: PLoS One. 2011 Sep 8;6(9):e24166. doi: 10.1371/journal.pone.0024166 (PMC3169588; doi:10.1371/journal.pone.0024166)
Supplement: Table S5 — Glomeromycota amplification on Medicago truncatula rhizosphere DNA extracts by real time Q-PCR in combination with FR1/FF390 primer set. (DOC) [file pone.0024166.s008.doc]

**Table S5. Glomeromycota amplification on *Medicago truncatula* rhizosphere DNA extracts by real time Q-PCR in combination with FR1/FF390 primer set**

| Sample Name | Target Name | Cт | Quantity |
| --- | --- | --- | --- |
| Sample 1 | ADN 18S | 22.41725 | 1.32E+06 |
| Sample 1 | ADN 18S | 22.45796 | 1.29E+06 |
| Sample 1 | ADN 18S | 22.51641 | 1.26E+06 |
| Sample 10 | ADN 18S | 21.62537 | 1.94E+06 |
| Sample 10 | ADN 18S | 21.75527 | 1.82E+06 |
| Sample 10 | ADN 18S | 21.77213 | 1.80E+06 |
| Sample 11 | ADN 18S | 22.22503 | 1.45E+06 |
| Sample 11 | ADN 18S | 22.22783 | 1.44E+06 |
| Sample 11 | ADN 18S | 22.26217 | 1.42E+06 |
| Sample 12 | ADN 18S | 21.91471 | 1.68E+06 |
| Sample 12 | ADN 18S | 21.97459 | 1.63E+06 |
| Sample 12 | ADN 18S | 21.9794 | 1.63E+06 |
| Sample 13 | ADN 18S | 21.57252 | 1.99E+06 |
| Sample 13 | ADN 18S | 21.59814 | 1.96E+06 |
| Sample 13 | ADN 18S | 21.63361 | 1.93E+06 |
| Sample 14 | ADN 18S | 22.10579 | 1.53E+06 |
| Sample 14 | ADN 18S | 22.245 | 1.43E+06 |
| Sample 14 | ADN 18S | 22.29843 | 1.40E+06 |
| Sample 15 | ADN 18S | 21.38755 | 2.17E+06 |
| Sample 15 | ADN 18S | 21.56496 | 1.99E+06 |
| Sample 15 | ADN 18S | 21.57417 | 1.98E+06 |
| Sample 2 | ADN 18S | 22.13583 | 1.51E+06 |
| Sample 2 | ADN 18S | 22.13847 | 1.51E+06 |
| Sample 2 | ADN 18S | 22.23389 | 1.44E+06 |
| Sample 3 | ADN 18S | 21.62617 | 1.93E+06 |
| Sample 3 | ADN 18S | 21.66189 | 1.90E+06 |
| Sample 3 | ADN 18S | 21.67661 | 1.89E+06 |
| Sample 4 | ADN 18S | 21.95655 | 1.65E+06 |
| Sample 4 | ADN 18S | 22.02356 | 1.60E+06 |
| Sample 4 | ADN 18S | 22.0347 | 1.59E+06 |
| Sample 5 | ADN 18S | 22.09807 | 1.54E+06 |
| Sample 5 | ADN 18S | 22.18315 | 1.48E+06 |
| Sample 5 | ADN 18S | 22.18501 | 1.47E+06 |
| Sample 6 | ADN 18S | 21.43838 | 2.12E+06 |
| Sample 6 | ADN 18S | 21.46772 | 2.09E+06 |
| Sample 6 | ADN 18S | 21.54229 | 2.02E+06 |
| Sample 7 | ADN 18S | 21.4717 | 2.09E+06 |
| Sample 7 | ADN 18S | 21.52257 | 2.03E+06 |
| Sample 7 | ADN 18S | 21.53305 | 2.02E+06 |
| Sample 8 | ADN 18S | 21.50995 | 2.05E+06 |
| Sample 8 | ADN 18S | 21.62926 | 1.93E+06 |
| Sample 8 | ADN 18S | 21.79895 | 1.78E+06 |
| Sample 9 | ADN 18S | 22.04129 | 1.58E+06 |
| Sample 9 | ADN 18S | 22.27629 | 1.41E+06 |
| Sample 9 | ADN 18S | 22.29609 | 1.40E+06 |
| Standard | ADN 18S | 14.75724 | 3.28E+07 |
| Standard | ADN 18S | 14.51765 | 3.28E+07 |
| Standard | ADN 18S | 14.34199 | 3.28E+07 |
| Standard | ADN 18S | 22.79301 | 3.28E+06 |
| Standard | ADN 18S | 23.11803 | 3.28E+06 |
| Standard | ADN 18S | 22.51408 | 3.28E+06 |
| Standard | ADN 18S | 31.92607 | 3.28E+04 |
| Standard | ADN 18S | 31.19095 | 3.28E+04 |
| Standard | ADN 18S | 31.42233 | 3.28E+04 |
| Standard | ADN 18S | 32.33016 | 3.28E+03 |
| Standard | ADN 18S | 32.24362 | 3.28E+03 |
| Standard | ADN 18S | 32.1924 | 3.28E+03 |
| Negative template | ADN 18S | 33.40814 | 6.32E+03 |
| Negative template | ADN 18S | 33.93155 | 4.90E+03 |
| Negative template | ADN 18S | 33.59558 | 5.77E+03 |
| Positive template | ADN 18S | 13.21063 | 1.15E+08 |
| Positive template | ADN 18S | 13.24704 | 1.13E+08 |
| Positive template | ADN 18S | 13.74064 | 8.92E+07 |
