# Supplementary material for: Validation and Application of a PCR Primer Set to Quantify Fungal Communities in the Soil Environment by Real-Time Quantitative PCR
Source: PLoS One. 2011 Sep 8;6(9):e24166. doi: 10.1371/journal.pone.0024166 (PMC3169588; doi:10.1371/journal.pone.0024166)
Supplement: Table S6 — Real-Time Q-PCR amplification results for the 5 soil samples used to test the specificity for fungi of FR1/FF390 primer set and to set up the template quantity in the real-time Q-PCR assay. NAN: Not A Number. (DOC) [file pone.0024166.s009.doc]

**Table S6. Real-Time Q-PCR amplification results for the 5 soil samples used to test the specificity for fungi of FR1/FF390 primer set and to set up the template quantity in the real-time Q-PCR assay.**

NAN: Not A Number

| **Sample Name** | **Template Quantity (ng)** | **Task** | **Ct** | **Quantity** |
| --- | --- | --- | --- | --- |
| 858 | 10 | Sample | 26.1 | 28534 |
| 858 | 10 | Sample | 26.1 | 28476 |
| 858 | 10 | Sample | 26.5 | 22618 |
| 858 | 5 | Sample | 26.9 | 16845 |
| 858 | 5 | Sample | 27.1 | 14753 |
| 858 | 5 | Sample | 27.7 | 10413 |
| 858 | 2.5 | Sample | 28.5 | 5833 |
| 858 | 2.5 | Sample | 28.6 | 5578 |
| 858 | 2.5 | Sample | 28.9 | 4724 |
| 858 | 1 | Sample | 29.8 | 2620 |
| 858 | 1 | Sample | 29.8 | 2576 |
| 858 | 1 | Sample | 30.1 | 2093 |
| 858 | 0.5 | Sample | 30.7 | 1434 |
| 858 | 0.5 | Sample | 30.9 | 1264 |
| 858 | 0.5 | Sample | 30.9 | 1249 |
| 1012 | 10 | Sample | 27.7 | 9959 |
| 1012 | 10 | Sample | 27.8 | 9310 |
| 1012 | 10 | Sample | 27.9 | 8993 |
| 1012 | 5 | Sample | 28.7 | 5279 |
| 1012 | 5 | Sample | 28.8 | 4990 |
| 1012 | 5 | Sample | 28.8 | 4980 |
| 1012 | 2.5 | Sample | 29.8 | 2608 |
| 1012 | 2.5 | Sample | 29.8 | 2578 |
| 1012 | 2.5 | Sample | 30.0 | 2212 |
| 1012 | 1 | Sample | 30.9 | 1236 |
| 1012 | 1 | Sample | 31.0 | 1172 |
| 1012 | 1 | Sample | 31.3 | 990 |
| 1012 | 0.5 | Sample | 31.8 | 716 |
| 1012 | 0.5 | Sample | 32.0 | 626 |
| 1012 | 0.5 | Sample | 32.2 | 536 |
| 1051 | 10 | Sample | 28.0 | 8455 |
| 1051 | 10 | Sample | 28.0 | 8169 |
| 1051 | 10 | Sample | 28.5 | 6196 |
| 1051 | 5 | Sample | 29.0 | 4412 |
| 1051 | 5 | Sample | 29.4 | 3313 |
| 1051 | 5 | Sample | 29.5 | 3192 |
| 1051 | 2.5 | Sample | 30.3 | 1825 |
| 1051 | 2.5 | Sample | 30.7 | 1448 |
| 1051 | 2.5 | Sample | 30.8 | 1357 |
| 1051 | 1 | Sample | 31.8 | 694 |
| 1051 | 1 | Sample | 31.9 | 667 |
| 1051 | 1 | Sample | 32.0 | 623 |
| 1051 | 0.5 | Sample | 32.1 | 595 |
| 1051 | 0.5 | Sample | 32.9 | 338 |
| 1051 | 0.5 | Sample | 33.4 | 244 |
| 1101 | 10 | Sample | 24.9 | 60948 |
| 1101 | 10 | Sample | 25.0 | 58063 |
| 1101 | 10 | Sample | 25.3 | 49353 |
| 1101 | 5 | Sample | 26.4 | 23925 |
| 1101 | 5 | Sample | 26.4 | 23593 |
| 1101 | 5 | Sample | 26.6 | 20720 |
| 1101 | 2.5 | Sample | 27.8 | 9735 |
| 1101 | 2.5 | Sample | 27.8 | 9651 |
| 1101 | 2.5 | Sample | 27.9 | 8794 |
| 1101 | 1 | Sample | 29.4 | 3303 |
| 1101 | 1 | Sample | 29.5 | 3149 |
| 1101 | 1 | Sample | 29.5 | 3087 |
| 1101 | 0.5 | Sample | 30.8 | 1315 |
| 1101 | 0.5 | Sample | 31.0 | 1224 |
| 1101 | 0.5 | Sample | 31.0 | 1178 |
| 1143 | 10 | Sample | 26.3 | 24802 |
| 1143 | 10 | Sample | 26.4 | 23777 |
| 1143 | 10 | Sample | 26.6 | 20113 |
| 1143 | 5 | Sample | 27.8 | 9568 |
| 1143 | 5 | Sample | 27.8 | 9295 |
| 1143 | 5 | Sample | 27.9 | 8699 |
| 1143 | 2.5 | Sample | 29.4 | 3341 |
| 1143 | 2.5 | Sample | 29.5 | 3137 |
| 1143 | 2.5 | Sample | 29.7 | 2772 |
| 1143 | 1 | Sample | 30.7 | 1401 |
| 1143 | 1 | Sample | 30.8 | 1335 |
| 1143 | 1 | Sample | 31.0 | 1221 |
| 1143 | 0.5 | Sample | 32.0 | 615 |
| 1143 | 0.5 | Sample | 32.1 | 596 |
| 1143 | 0.5 | Sample | 32.5 | 448 |
| NTC | 0 | Negative Template | NAN | 0 |
| NTC | 0 | Negative Template | NAN | 0 |
| st | NAN | Standard | 14.6 | 32800000 |
| st | NAN | Standard | 18.5 | 3280000 |
| st | NAN | Standard | 19.3 | 3280000 |
| st | NAN | Standard | 22.7 | 328000 |
| st | NAN | Standard | 22.9 | 328000 |
| st | NAN | Standard | 28.9 | 3280 |
| st | NAN | Standard | 29.5 | 3280 |
| T+ | 2 | Positive template | 24.4 | 85652 |
| T+ | 2 | Positive template | 24.2 | 96599 |
