# Supplementary material for: Validation and Application of a PCR Primer Set to Quantify Fungal Communities in the Soil Environment by Real-Time Quantitative PCR
Source: PLoS One. 2011 Sep 8;6(9):e24166. doi: 10.1371/journal.pone.0024166 (PMC3169588; doi:10.1371/journal.pone.0024166)
Supplement: Table S7 — Real-Time Q-PCR amplification results for the 24 soil samples used for the ecological validation of real-time Q PCR in combination with FR1/FF390 primer set. NAN: Not A Number. (DOC) [file pone.0024166.s010.doc]

**Table S7. Real-Time Q-PCR amplification results for the 24 soil samples used for the ecological validation of real-time Q PCR in combination with FR1/FF390 primer set.**

NAN: Not A Number

| Sample Name | Template quantity (ng) | Task | Ct | Quantity |
| --- | --- | --- | --- | --- |
| 1004 | 2.5 | Sample | 25.8 | 13948 |
| 1004 | 2.5 | Sample | 23.4 | 68938 |
| 1004 | 2.5 | Sample | 25.3 | 18796 |
| 1053 | 2.5 | Sample | 24.5 | 32791 |
| 1053 | 2.5 | Sample | 23.1 | 82798 |
| 1095 | 2.5 | Sample | 25.0 | 23092 |
| 1095 | 2.5 | Sample | 22.9 | 96554 |
| 1095 | 2.5 | Sample | 24.6 | 29864 |
| 1099 | 2.5 | Sample | 25.1 | 22751 |
| 1099 | 2.5 | Sample | 23.6 | 57876 |
| 1099 | 2.5 | Sample | 24.8 | 27572 |
| 1146 | 2.5 | Sample | 26.8 | 7434 |
| 1146 | 2.5 | Sample | 25.7 | 14552 |
| 1146 | 2.5 | Sample | 26.1 | 11417 |
| 1182 | 2.5 | Sample | 25.2 | 21344 |
| 1182 | 2.5 | Sample | 25.5 | 17109 |
| 1182 | 2.5 | Sample | 24.9 | 25698 |
| 1220 | 2.5 | Sample | 28.6 | 2184 |
| 1220 | 2.5 | Sample | 28.1 | 3154 |
| 1220 | 2.5 | Sample | 27.8 | 3796 |
| 1224 | 2.5 | Sample | 24.9 | 25488 |
| 1224 | 2.5 | Sample | 25.2 | 21095 |
| 1224 | 2.5 | Sample | 25.0 | 24143 |
| 1305 | 2.5 | Sample | 26.0 | 12521 |
| 1305 | 2.5 | Sample | 25.5 | 16470 |
| 1305 | 2.5 | Sample | 25.7 | 15265 |
| 633 | 2.5 | Sample | 23.7 | 56344 |
| 633 | 2.5 | Sample | 24.8 | 27121 |
| 633 | 2.5 | Sample | 23.9 | 47344 |
| 634 | 2.5 | Sample | 24.5 | 32774 |
| 634 | 2.5 | Sample | 24.7 | 28680 |
| 634 | 2.5 | Sample | 24.5 | 32502 |
| 693 | 2.5 | Sample | 25.9 | 13084 |
| 693 | 2.5 | Sample | 22.8 | 98830 |
| 693 | 2.5 | Sample | 26.0 | 12406 |
| 750 | 2.5 | Sample | 23.1 | 82471 |
| 750 | 2.5 | Sample | 22.9 | 91523 |
| 807 | 2.5 | Sample | 25.0 | 23571 |
| 807 | 2.5 | Sample | 22.7 | 106771 |
| 807 | 2.5 | Sample | 24.9 | 24751 |
| 810 | 2.5 | Sample | 22.6 | 111658 |
| 810 | 2.5 | Sample | 22.1 | 159143 |
| 810 | 2.5 | Sample | 22.8 | 103022 |
| 854 | 2.5 | Sample | 24.3 | 36713 |
| 854 | 2.5 | Sample | 23.3 | 71731 |
| 854 | 2.5 | Sample | 23.8 | 53226 |
| 857 | 2.5 | Sample | 24.5 | 32824 |
| 857 | 2.5 | Sample | 24.3 | 36322 |
| 857 | 2.5 | Sample | 25.1 | 22046 |
| 907 | 2.5 | Sample | 26.1 | 11686 |
| 907 | 2.5 | Sample | 26.1 | 11675 |
| 907 | 2.5 | Sample | 25.1 | 22571 |
| 910 | 2.5 | Sample | 24.3 | 38341 |
| 910 | 2.5 | Sample | 22.2 | 149657 |
| 910 | 2.5 | Sample | 24.5 | 32675 |
| 914 | 2.5 | Sample | 25.5 | 16751 |
| 914 | 2.5 | Sample | 24.8 | 27265 |
| 914 | 2.5 | Sample | 25.2 | 20695 |
| 917 | 2.5 | Sample | 23.7 | 56397 |
| 917 | 2.5 | Sample | 23.6 | 57676 |
| 917 | 2.5 | Sample | 23.6 | 58166 |
| 963 | 2.5 | Sample | 25.2 | 20667 |
| 963 | 2.5 | Sample | 23.1 | 79605 |
| 963 | 2.5 | Sample | 24.8 | 26424 |
| 965 | 2.5 | Sample | 25.7 | 14578 |
| 965 | 2.5 | Sample | 23.9 | 49158 |
| 965 | 2.5 | Sample | 25.4 | 18625 |
| 968 | 2.5 | Sample | 22.6 | 111538 |
| 968 | 2.5 | Sample | 22.7 | 109815 |
| 968 | 2.5 | Sample | 22.5 | 119051 |
| NTC | 0 | Negative template | 34.9 | 35 |
| NTC | 0 | Negative template | 34.2 | 58 |
| T+ | 2 | Positive template | 20.0 | 617108 |
| st | NAN | Standard | 14.2 | 32800000 |
| st | NAN | Standard | 14.2 | 32800000 |
| st | NAN | Standard | 17.5 | 3280000 |
| st | NAN | Standard | 17.5 | 3280000 |
| st | NAN | Standard | 20.7 | 328000 |
| st | NAN | Standard | 20.9 | 328000 |
| st | NAN | Standard | 24.3 | 32800 |
| st | NAN | Standard | 24.3 | 32800 |
| st | NAN | Standard | 27.8 | 3280 |
| st | NAN | Standard | 27.6 | 3280 |
| st | NAN | Standard | 31.8 | 328 |
| st | NAN | Standard | 32.0 | 328 |
